# Characterization of distinct circular RNA signatures in solid tumors

**DOI:** 10.1186/s12943-022-01546-4

**Published:** 2022-03-02

**Authors:** Chengdi Wang, Wen-Rong Liu, Shuangyan Tan, Jian-Kang Zhou, Xiaomin Xu, Yue Ming, Jian Cheng, Jiao Li, Zhen Zeng, Yuanli Zuo, Juan He, Yong Peng, Weimin Li

**Affiliations:** Department of Respiratory and Critical Care Medicine, Laboratory of Molecular Oncology, Frontiers Science Center for Disease-related Molecular Network, Med-X Center for Manufacturing, National Clinical Research Center for Geriatrics, State Key Laboratory of Biotherapy, West China Hospital, Sichuan University, Chengdu, 610041 China

**Keywords:** Circular RNA, Solid tumors, Transcriptome sequencing, CircLIFR

## Abstract

**Background:**

Circular RNAs (circRNAs) are differentially expressed between normal and cancerous tissues, contributing to tumor initiation and progression. However, comprehensive landscape of dysregulated circRNAs across cancer types remains unclear.

**Methods:**

In this study, we conducted Ribo-Zero transcriptome sequencing on tumor tissues and their adjacent normal samples including glioblastoma, esophageal squamous cell carcinoma, lung adenocarcinoma, thyroid cancer, colorectal cancer, gastric cancer and hepatocellular carcinoma. CIRCexplorer2 was employed to identify circRNAs and dysregulated circRNAs and genes were determined by DESeq2 package. The expression of hsa_circ_0072309 (circLIFR) was measured by reverse transcription and quantitative real-time PCR, and its effect on cell migration was examined by Transwell and wound healing assays. The role of circLIFR in tumor metastasis was evaluated via mouse models of tail-vein injection and spleen injection for lung and liver metastasis, respectively.

**Results:**

Distinct circRNA expression signatures were identified among seven types of solid tumors, and the dysregulated circRNAs exhibited cancer-specific expression or shared common expression signatures across cancers. Bioinformatics analyses indicated that aberrant expression of host genes and/or RNA-binding proteins contributed to circRNA dysregulation in cancer. Finally, circLIFR was experimentally validated to be downregulated in six solid tumors and to significantly inhibit cell migration in vitro and tumor metastasis in vivo.

**Conclusions:**

Our results provide a comprehensive landscape of differentially expressed circRNAs in solid tumors and highlight that circRNAs are extensively involved in cancer pathogenesis.

**Supplementary Information:**

The online version contains supplementary material available at 10.1186/s12943-022-01546-4.

## Background

Circular RNAs (circRNAs) are a class of single-stranded endogenous RNAs with covalently closed-loop structures generated by back-splicing [1]. According to the origin of sequences, circRNAs are classified into three major groups, namely exonic circRNA (EcRNA), exon-intron circRNA (EIciRNA) and intronic circRNA (ciRNA). The biogenesis of circRNA is tightly regulated by *cis*-acting elements and *trans*-acting proteins. Moreover, a single gene locus could produce multiple circRNA isoforms by alternative circularization [2]. Recently, circRNAs have emerged as essential regulators in various biological processes through diverse mechanisms such as regulating transcription, sponging microRNA (miRNA), acting as RNA-binding protein (RBP) decoy, and translating into protein [1, 3].

Increasing evidence has revealed that circRNAs are commonly dysregulated in cancer and exert crucial functions during tumor initiation and development. For instance, circ-SMO is significantly upregulated in glioblastoma (GBM) and activates the Hedgehog pathway to promote tumorigenesis through encoding a novel protein SMO-193a.a. [4]. In addition, epigenetic silencing of the circRNA CDR1as in melanoma was demonstrated to promote invasion in vitro and metastasis in vivo via an IGF2BP3-mediated mechanism [5]. To identify cancer-related circRNAs, Ruan et al characterized the expression landscape of circRNAs across ~ 1000 human cancer cell lines from CCLE (Cancer Cell Line Encyclopedia) polyA-enriched transcriptome sequencing (RNA-seq) data [6], but the polyA-enriched method has bias and loses some circRNAs. In addition, Chinnaiyan group employed an exome capture RNA sequencing protocol to detect and characterize circRNAs across more than 10 types of tumor samples, in which only prostate tumors had paired adjacent normal tissues [7]. Therefore, dysregulated circRNAs that show consistent or discordant alterations across different cancer types remain elusive.

To address this question, we performed rRNA-depleted RNA-seq on 122 tumors and their matched normal samples across seven types of malignancies including GBM, esophageal squamous cell carcinoma (ESCC), lung adenocarcinoma (LUAD), thyroid cancer (THCA), colorectal cancer (CRC), gastric cancer (GC) and hepatocellular carcinoma (HCC). First, we characterized the tissue-specific expression patterns of circRNAs in normal tissues and identified dysregulated circRNA expression signatures across cancer types. Moreover, the tissue-specific expression of circRNAs and the dysregulated circRNAs resulted from the expression of their host genes or RBPs. Finally, we validated that the commonly downregulated circLIFR inhibited cell invasion in vitro and metastasis in vivo, highlighting its tumor-suppressive role in solid tumors.

## Materials and methods

### Clinical sample collection

This study was approved by the Ethics Committee of West China Hospital of Sichuan University and written informed consent was obtained from patients. Samples were collected following the relevant ethical regulations for human participants. A total of 122 pairs of fresh tumors and their matched normal tissues from seven types of malignancies (Additional file 1: Table S1) were collected from West China Hospital to conduct RNA-seq. Another 90 tumors and their adjacent normal samples were collected for validation by quantitative real-time PCR.

### Transcriptome sequencing

Total RNAs were isolated from patient tissues with TRIzol™ reagent (Invitrogen, USA) according to the manufacturer’s protocol. The qualified RNA samples were treated with an Epicentre Ribo-zero™ rRNA Removal Kit (Epicentre Technologies, USA) to remove ribosomal RNA (rRNA), and a strand-specific sequencing library was constructed using the NEBNext® UltraTM Directional RNA Library Prep Kit for Illumina® (NEB, USA). Paired-end sequencing was performed using Illumina HiSeq X Ten platform for paired-end 150 bp (PE150) reads.

### CircRNA identification and quantification

The quality of raw reads was evaluated with FastQC (v0.11.8). Reads were trimmed of adapter sequences and low-quality bases with Trimmomatic (v0.36) [8]. The passed clean reads were aligned to the GRCh37.p13 reference genome (GRCh37.p13). The aligner STAR (v2.5.3a) [9] was used for alignment with the following settings: chimSegmentMin 10. To annotate circRNAs to genes, we employed Circexplorer2 (v2.3.8) [2] on the junction files generated by the above chimeric aligning step. CircRNAs annotated in the circBase [10] were subjected to further analysis.

To allow direct comparison between samples, circRNAs in each sample were normalized as the number of back-spliced RPMs (reads per million mapped reads) [11]. In addition, the junction ratio [12] was calculated as the ratio of back-spliced reads versus forward-spliced reads. A junction ratio over 0.5 indicated that the circRNA was expressed at higher levels than its linear counterpart. Pearson correlation analysis was performed to quantify the similarities of expression profiles between circRNAs and their corresponding host genes.

### Transcriptome analysis

The clean reads were mapped to the human genome (GRCh37.p13) using HISAT2 (v2.1.0) [13]. StringTie (v2.0.4) [14] was employed to estimate gene abundance with the guidance of reference gene annotation (GENCODE v19). Gene expression levels measured as fragments per kilobase per million (FPKM) were extracted from the output of StringTie. The number of reads mapped on each gene was counted using the preDE.py script provided by StringTie.

### Tissue specificity ananlysis

To explore the tissue specificity of circRNAs, we filtered circRNAs with median RPM ≥ 0.1 in at least one tissue for further analysis. The median RPM of a circRNA was used to calculate the tissue specificity score of this circRNA [15]. The tissue specificity index (TSI) was calculated using the following equation:

$$\mathrm{TSI}=\frac{\sum_{i=1}^n\left(1-\frac{x_i}{x_{max}}\right)}{n-1}$$

Where n denotes the number of measured tissues, *x*_*i*_ represents the median RPM expression of circRNA in the tissue, and *x*_*max*_ was the maximal expression of circRNA across all the tissues.

Likewise, for characterization of the tissue specificity of protein-coding genes, genes with median FPKM ≥10 in at least one tissue were retained and then the TSI of genes was calculated. Using the threshold suggested in a previous study [16], circRNAs or genes were categorized into ubiquitous (TSI ≤ 0.3) and tissue-specific (TSI ≥ 0.85) categories.

### Differential expression and functional enrichment analysis

The R package DESeq2 [17] was used for differential expression analysis. For patients’ samples, all protein-coding genes were kept for analysis, while only circRNAs with RPM ≥ 0.1 in at least 10% of patients’ samples were retained for differential expression analysis. Genes and circRNAs with fold-change greater than 2 and adjusted *p* value less than 0.05 were determined to be significantly differentially expressed. GO (Gene Ontology), KEGG (Kyoto Encyclopedia of Genes and Genomes) and GSEA (Gene Set Enrichment Analysis) were conducted using the clusterProfiler [18] package.

### Identification of putative RBPs involved in the regulation of circRNA expression

The comprehensive information of RBPs was obtained from Gebauer’s research [19]. RBPs were annotated with GO database and those involved in biological processes such as RNA splicing, helicase, endonuclease and deaminase (Additional file 2: Table S2) [6] were selected to analyze their contributions to circRNA biogenesis. To identify putative RBPs regulating circRNA expression, we calculated the Pearson correlation coefficients between circRNAs and RBPs according to their expression profiles. The circRNA-RBP pairs with coefficiencies ≥0.7 were regarded as strong correlations [20]. The eCLIP-seq data of RBFOX1 and QKI were downloaded from the NCBI Gene Expression Omnibus (accession number GSE98210), and the binding sites of RBFOX1 and QKI were obtained from a previous study [21].

### RT-PCR and quantitative real-time PCR

Total RNAs were reversely transcribed using PrimeScript™ RT Reagent Kit with gDNA Eraser (TAKARA). To identify the back-spliced junction site of circRNA, the resultant cDNAs were subjected to PCR reactions using Phanta® Max Super-Fidelity DNA Polymerase (Vazyme) and specific divergent primers, and the PCR products were purified for Sanger sequencing. For circRNA quantification, quantitative real-time PCR was performed with specific divergent primers spanning the junction site using TB Green® Premix Ex Taq™ II (Tli RNase H Plus) (TAKARA) on a StepOne Plus real-time PCR system (Applied Biosystems).U6, β-actin or vinculin mRNA was used as a control for normalization. The primers are listed in Additional file 3: Table S3.

### Plasmid construction

The plasmid pcDNA3.1 (+) Laccase2 MCS Exon was generously provided by Prof. Jeremy E. Wilusz (University of Pennsylvania) [22], and its Laccase2 flanking sequences were amplified and inserted into pCDH-EF1-MCS-EF1-Puro at *EcoR*I and *BamH*I sites to obtain the plasmid pCDH-EF1-Laccase2-MCS-Puro. To construct the circLIFR-overexpressing plasmid, the full-length sequence of circLIFR was amplified with cDNAs from KYSE30 cells, and inserted into the vector pCDH--EF1-Laccase2-MCS-Puro at *Age*I site. The primers used for cloning are listed in Additional file 3: Table S3.

### Cell culture and construction of stable cells

The human esophageal squamous cells KYSE150 and KYSE30 were cultured in RPMI-1640 medium (Gibco), while the hepatocellular carcinoma cell SK-Hep-1 and the colorectal cancer cell HCT-116 were cultured in DMEM medium (Gibco), supplemented with 10% FBS and 1% penicillin/streptomycin (HyClone) at 37 °C in a humidified 5% CO_2_ incubator. For lentivirus preparation, HEK-293 T cells were transfected with lentivirus expressing plasmid plus the packaging plasmid pCMV-dR8.2 dvpr and the envelope plasmid pCMV-VSVG. Then the resultant lentiviruses infected cells to construct stable cells by puromycin selection.

### Fluorescence in situ hybridization (FISH) assay

The fluorescence-labeled probe for circLIFR was designed spanning the junction site and synthesized by Sangon Biotech. FISH experiments were performed using Ribo™ Fluorescent In Situ Hybridization Kit (RiboBio) according to the manufacturer’s instructions. Briefly, cells were fixed in 4% paraformaldehyde for 10 min, permeabilized by 0.5% Triton X-100 at 4 °C for 5 min, prehybridized at 37 °C for 30 min, and then hybridized in situ at 37 °C overnight with fluorescence-labeled probes. After extensive washing, nuclei were stained with DAPI (4′,6-diamidino-2-phenylindole). Images were acquired on an Olympus Fluoview laser scanning confocal microscope. The probe sequences are listed in Additional file 3: Table S3.

### Cytoplasmic/Nuclear fractionation

Cells were resuspended in RLN buffer (50 mM Tris-HCl, pH 7.0, 140 mM NaCl, 1.5 mM MgCl_2_, 0.5% NP-40) and incubated on ice for 1 min. Then the cell lysates were centrifuged at 300 g for 3 min to collect the supernatants as the cytoplasmic fractions. The remaining pellets were washed with RLN buffer twice and resuspended in RLN buffer again as nuclear fractions.

### Cellular function assays

For migration assay, cells were suspended in RPMI-1640 medium containing 5% BSA and seeded into the top chamber (Corning), and RPMI-1640 medium supplemented with 10% FBS was added into the bottom chambers. After incubation for a certain time, the migrated cells were fixed with 4% paraformaldehyde for 20 min, stained with 1% crystal violet (Sigma) for 15 min and counted under an inverted phase-contrast microscope. For wound healing assay, cells were cultured in 6-well plates and scratched directly using a sterilized pipette tip. After washing with physiological saline gently, cells were continued to culture for a certain time in RPMI-1640 or DMEM medium containing 0.1% FBS.

### Western blot analysis

Proteins were separated on 8% SDS-PAGE and transferred onto PVDF membranes (Millipore). After blocking with 5% nonfat dry milk, membranes were incubated with primary antibodies (anti-Lamin A/C and anti-β-actin, 1:1000 dilution, Cell Signaling Technology) overnight at 4 °C. Followed by incubation with secondary antibody for 1 h at room temperature and washing, the immunoreactive signals were visualized by an enhanced chemiluminescence kit (Thermo Fisher Scientific) and detected by a Bio-Rad ChemiDocTM XRS^+^ Imaging System with lmage Lab™ Software.

### Animal experiments

All mouse procedures were approved by the Institutional Animal Care and Use of West China Hospital, Sichuan University. BALB/c nude mice were purchased from Beijing HFK Bioscience Co. Ltd. For lung metastasis, 1 × 10^6^ KYSE30 cells suspended in 200 μl PBS were injected into the tail vein. Six weeks later, mice were sacrificed to observe the foci of lung by H&E staining. For liver metastasis, 6 × 10^5^ SK-Hep-1 cells suspended in 100 μl PBS were injected into the spleen. Eight weeks later, mice were sacrificed to examine the foci in livers by H&E staining. For imaging tumors in live animals, mice were anaesthetized with isoflurane and injected intraperitoneally with 100 μl of VivoGlo™ luciferin (Biovision) solution (15 mg/ml). 15 min later, images were acquired with the Xenogen IVIS Lumina series II.

### Statistical analysis

Data are shown as the mean ± standard error of the mean (SEM) and are representative of at least three independent experiments. Statistical analyses were performed using Prism software (GraphPad Software 8). All significant differences were calculated using two-tailed Student’s *t*-test, one-way analysis of variance or Wilcoxon rank-sum test, and *p *value less than 0.05 was considered statistically significant. All visualizations were conducted in the R environment (v4.0.3) (http://www.r-project.org/).

## Results

### Characteristics of circRNAs in the human transcriptome

**Fig. 1:**
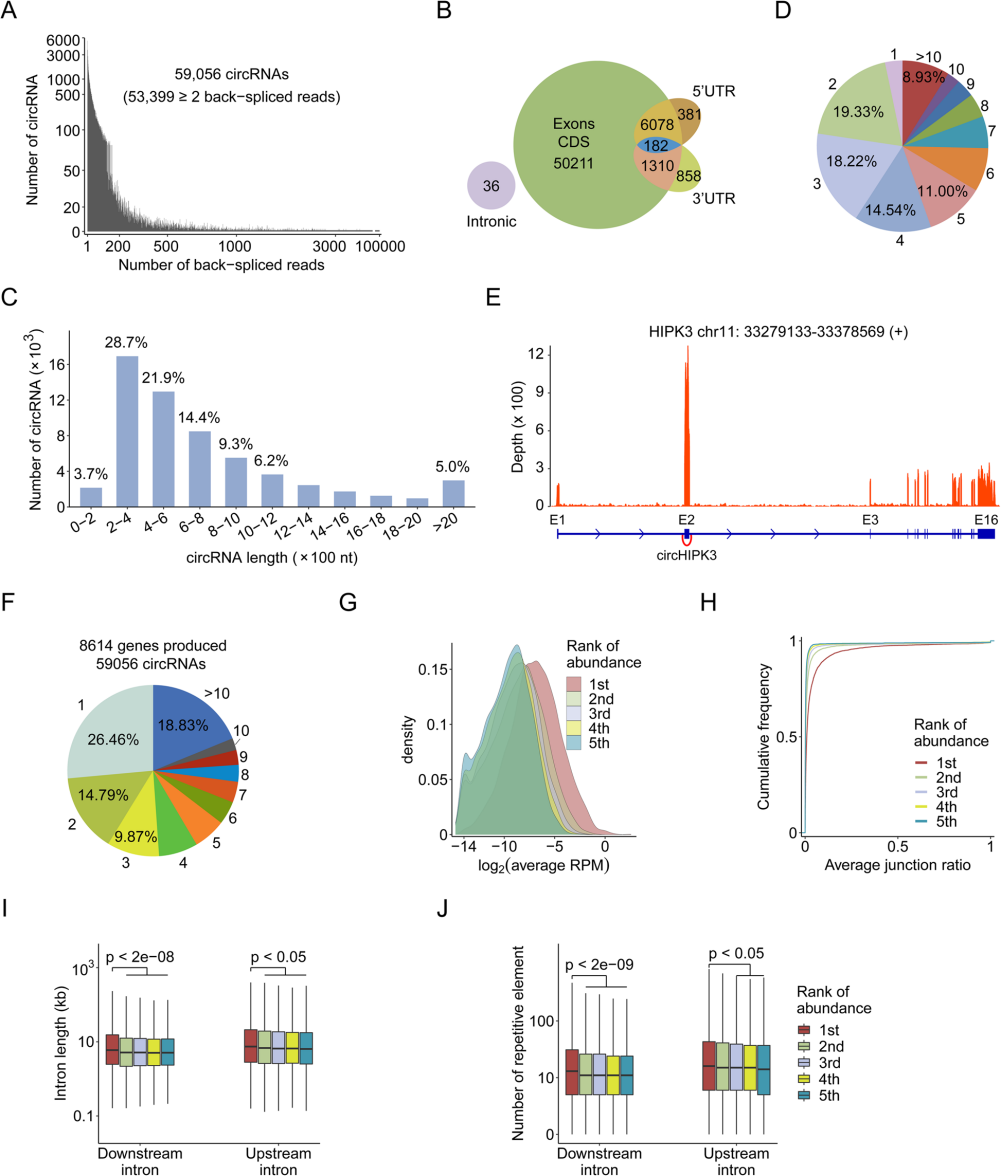
Characteristics of circRNAs identified in our Ribo-zero sequencing data. **A** The distribution of the number of back-spliced reads of circRNAs. **B** The genomic origin of circRNAs. **C** The distribution of putative spliced lengths of exonic circRNAs. **D** The distribution of exon number of exonic circRNAs. **E** Reads coverage of circHIPK3 in the GBM_P1T sample. This circRNA was more highly expressed than its cognate linear transcript. **F** The number of circRNAs produced per host gene. **G**-**J** Comparison of circRNA average RPM expression (**G**), average junction ratio (**H**), flanking intron length (**I**) and repetitive element count (**J**) of the top five abundantly expressed circRNA isoforms derived from one gene locus. The *p*-value was calculated using the Wilcoxon rank-sum test

To comprehensively explore the circRNA landscape in solid tumors, 122 tumor samples across seven types of malignancies were collected to conduct strand-specific and rRNA-depleted RNA sequencing together with their corresponding noncancerous normal tissues (Additional file 1: Table S1). We used CIRCexplorer2 to identify a total of 59,056 circRNAs annotated in the circBase, of which 53,399 (90.42%) were supported by at least two back-spliced junction reads (Fig. 1A). According to genomic region, the majority of circRNAs (59,020; 99.94%) were derived from exonic regions including protein coding sequences (CDSs) and UTRs (untranslated regions) (Fig. 1B). Interestingly, 182 circRNAs harbored complete CDS, as they originated from both the CDS and UTRs, which implied their potential for translating into intact peptides. These exonic circRNAs were 200–1200 nt in length and mainly consisted of 2–5 exons (Fig. 1C-D, Additional file 3: Table S4 and S5). Interestingly, 22 circRNAs were found to exhibit much higher expression than their cognate linear transcripts (Additional file 3: Table S6), suggesting their important roles in cellular functions. For instance, circHIPK3, an oncogenic circRNA derived from exon 2 of the HIPK3 gene locus [23], showed significantly higher expression than its linear counterpart (Fig. 1E).

Given the alternative back-splicing mechanism, we explored the circRNA diversity and found nearly 73.54% of host genes could produce at least 2 circRNA isoforms (Fig. 1F and Additional file 3: Table S7). Strikingly, 18.83% of host genes could give rise to more than 10 circRNA isoforms. Moreover, genes were found to show a tendency to express a predominant circRNA isoform (Fig. 1G-H). Accordingly, the introns flanking circularized exons of the predominant circRNA isoform were much longer and had more repetitive elements (Fig. 1I-J), suggesting that the sequence structures within introns might facilitate the biogenesis of these circRNAs.

### Landscape of tissue-specific circRNAs

To characterize the tissue-specific circRNAs, protein-coding genes were initially depicted to demonstrate the reliability of our RNA-seq data. We considered the genes with high abundance (median FPKM expression value ≥10 in at least one tissue) and quantified the tissue specificity of each gene with TSI. In total, 1590 genes with TSI ≤ 0.3 were determined to be ubiquitous across the tissues (Additional file 4: Fig. S1A and Additional file 5: Table S8), of which 1042 (66.92%) genes (Additional file 5: Table S8, marked in red) were reported as housekeeping genes by a previous study [24], suggesting their potential functions in the maintenance of basal cellular processes. Moreover, these ubiquitous genes were predominantly enriched in biological processes and signaling pathways that are fundamental to cells, such as RNA splicing and transporting (Additional file 4: Fig. S1B-C). We also identified 1765 tissue-specific protein-coding genes (TSI ≥ 0.85, Additional file 4: Fig. S1D and Additional file 6: Table S9) and further confirmed their gene expression profiles in normal tissue from the GTEx database (Additional file 4: Fig. S2). Functional enrichment analysis of these tissue-specific genes demonstrated tissue-specific biological processes and signaling pathways (Additional file 4: Fig. S1E-F and Fig. S3). For instance, the genes specific to the brain were enriched in brain-specific biological processes and signaling pathways, such as synapse, learning, cognition and axon guidance (Additional file 4: Fig. S1E-F). Collectively, these results confirmed the reliability of our sample preparation and data analysis.

**Fig. 2:**
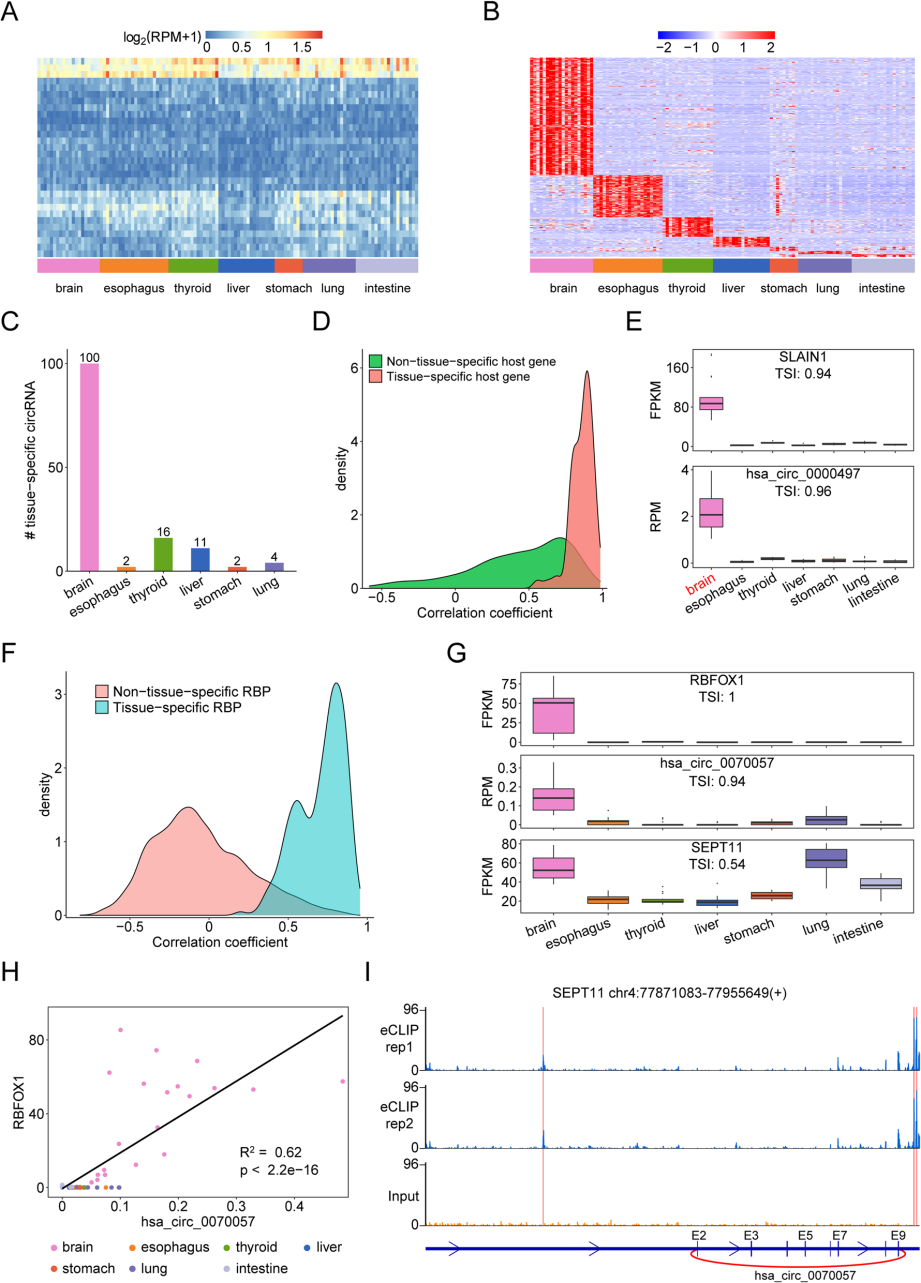
Tissue-specific expression patterns of circRNAs in human normal tissues. A Heatmap of ubiquitously expressed circRNAs. **B** Heatmap of tissue-specifically expressed circRNAs. **C** The number of circRNAs produced from tissue-specific genes. **D** The Pearson correlation coefficients of tissue-specific circRNAs with host gene expression. **E** The tissue specificity of hsa_circ_0000497 was governed by the brain-specific host gene SLAIN1. **F** The Pearson correlation coefficients of tissue-specific circRNAs with RBP expression. **G** Expression of RBFOX1, hsa_circ_0070057 and its host gene SEPT11 in different tissues. **H** Correlation of hsa_circ_0070057 and RBFOX1 expression. **I** eCLIP-seq read density for RBFOX1 within introns flanking the hsa_circ_0070057 back-splicing site. The vertical red lines indicate the GCATG binding motifs of RBFOX1

Considering that most circRNAs were discovered at low frequency and expressed at low levels, 1762 circRNAs with high abundance (median RPM expression value ≥0.1 in at least one tissue) were retained to obtain a high-confidence profile. As a result, 30 circRNAs with TSI ≤ 0.3 were defined as the ubiquitous circRNAs (Fig. 2A and Additional file 7: Table S10), indicating their important functions in fundamental cellular processes. In addition, we found 439 circRNAs with TSI ≥ 0.85 and defined them as tissue-specific circRNAs (Fig. 2B and Additional file 8: Table S11), implying their tissue-specific functions. Moreover, we found that the brain exhibited the highest number of tissue-specific circRNAs while the intestine exhibited the least (Fig. 2B and Additional file 8: Table S11). For instance, we identified that hsa_circ_0000497 was exclusively highly expressed in the brain (Fig. 2E), which was further supported by circAtlas data (Additional file 4: Fig. S4). However, the functions of these ubiquitous and tissue-specific circRNAs remain elusive.

### Tissue-specific expression of host genes and/or RBPs contributes to the tissue specificity of circRNAs

The tissue-specific expression signatures of circRNAs may be the consequence of their biogenesis. Given that most circRNAs were processed from the primary transcripts transcribed from gene loci, we initially examined whether both circRNA and its host gene showed the same tissue specificity and found that only 135 (30.75%) circRNAs were produced from tissue-specific host genes (Fig. 2C and Additional file 4: Fig. S5A). Pearson correlation analysis further supported that circRNAs derived from tissue-specific genes exhibited a strong correlation with their host genes (Fig. 2D), implying that the expression of these circRNAs highly depend on their host gene expression. For example, SLIAN1 is a microtubule plus-end tracking protein exclusively expressed in the brain to regulate microtubule dynamics and promote axonal development [25], so hsa_circ_0000497 derived from the SLAIN1 gene was also highly expressed in the brain (Fig. 2E and Additional file 4: Fig. S5B-D). Therefore, tissue-specific expression of host genes contributes to the tissue specificity of these tissue-specific circRNAs.

It was worth noting that 304 (69.25%) tissue-specific circRNAs were derived from nontissue-specific genes, implying that other factors contribute to their tissue specificity. As RBPs are known to play a role in circRNA biogenesis, we speculated that tissue-specific RBPs may regulate the tissue specificity of circRNAs. According to a previous study [6], 215 RBPs (Additional file 2: Table S2) were selected to calculate their TSI, and 8 RBPs (Additional file 3: Table S12) with TSI ≥ 0.85 were identified as tissue-specific. Next, we analyzed correlations between 304 circRNAs and 8 RBPs, and found that 155 (35.31%) circRNAs showed a strong correlation with these RBPs (Pearson correlation coefficient ≥ 0.7, Fig. 2F), indicating that the tissue specificity of these circRNAs may be regulated by tissue-specific RBPs. Among these RBPs, RBFOX1 was found to be exclusively expressed in the brain (TSI = 1) (Fig. 2G), consistent with a previous study [26]. Therefore, we chose RBFOX1 as an example to investigate its potential regulatory mechanisms on the biogenesis of tissue-specific circRNAs. First, we found that brain-specific hsa_circ_0070057 had a strong association with RBFOX1 expression in the brain, not with the expression of its host gene (Fig. 2G-H), Furthermore, eCLIP-seq data of RBFOX1 [21] demonstrated that RBFOX1 indeed bound to the flanking introns of hsa_circ_0070057 (Fig. 2I), suggesting that RBFOX1 facilitates the biogenesis of this circRNA. In addition, brain-specific hsa_circ_0073539 may also be regulated by QKI (TSI = 0.86 in brain) which is a well-known factor in circRNA biogenesis (Additional file 4: Fig. S6) [27], not by its host gene MAN2A1. Together, tissue-specific expression of RBPs may play an important role in the biogenesis of tissue-specific circRNAs.

### Expression patterns of differentially expressed circRNAs in cancer

**Fig. 3:**
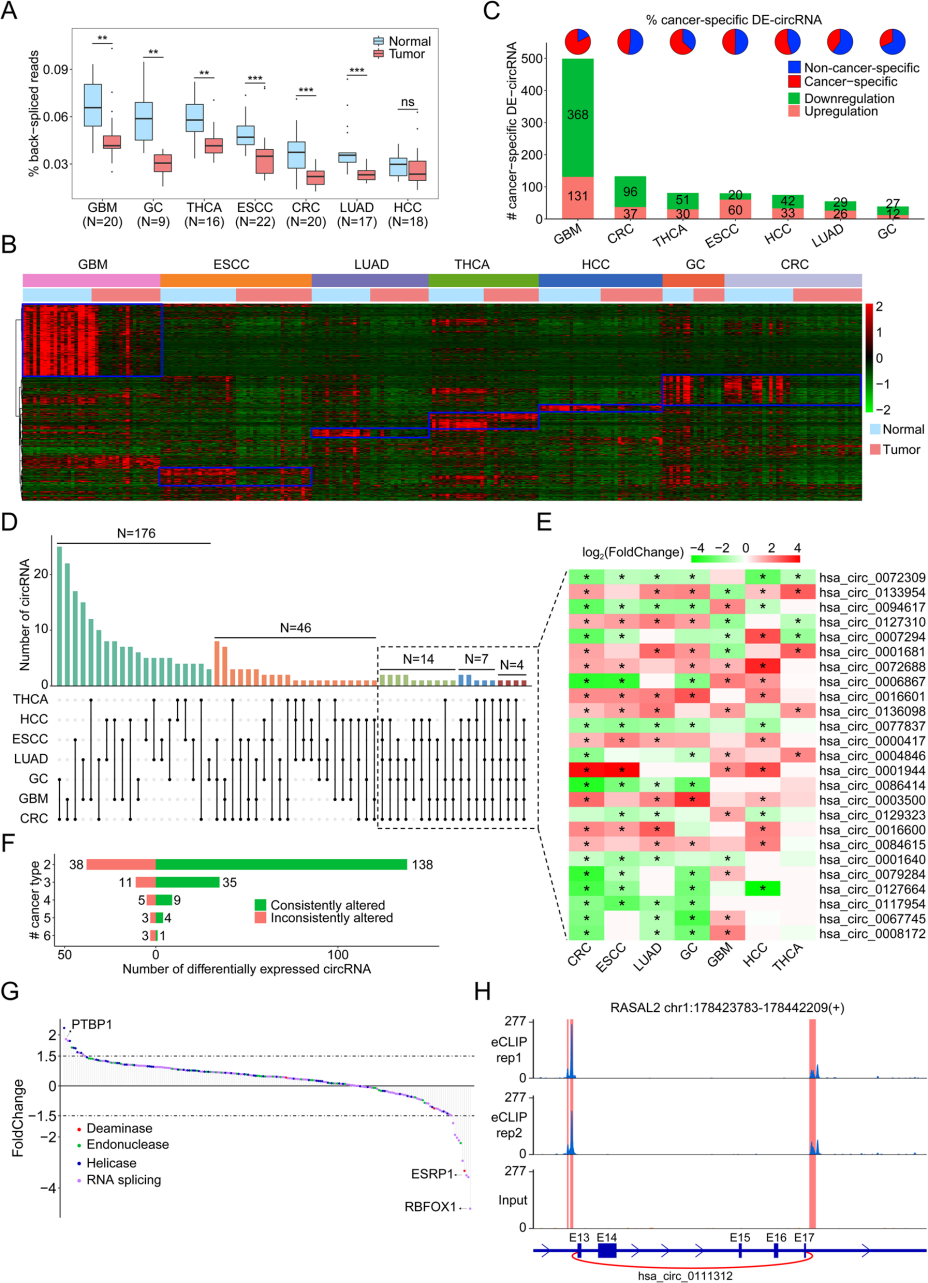
Characterization of differentially expressed circRNAs in seven solid tumors. **A** Total circRNA abundance in tested cancers. **B** Profiles of differentially expressed circRNAs identified in this study. The blue box indicates the unique circRNA expression signatures of each cancer. **C** The number of cancer-specific differentially expressed circRNAs. **D** List of differentially expressed circRNAs shared by at least two types of cancers. **E** Heatmap of twenty-five differentially expressed circRNAs shared by at least four types of cancers. The asterisk indicates circRNA expression is significantly altered. Red and green colors indicate upregulation and downregulation, respectively. **F** The number of differentially expressed circRNAs that exhibited consistent or inconsistent expression changes in at least two cancer types. **G** Expression alteration of RBPs associated with circRNA biogenesis in GBM. **H** eCLIP-seq read density for RBFOX1 within introns flanking hsa_circ_0111312 back-splicing site. The vertical red box indicates the GCATG binding motifs of RBFOX1

Emerging evidence has reported that circRNAs are frequently dysregulated in cancer. To depict the abnormal expression of circRNAs in cancer, we compared the global circRNA abundance in tumors and their paired normal samples, and found the downregulation of global circRNA abundance in almost all cancer types (Fig. 3A) under the comparable sequencing depths (Additional file 4: Fig. S7A and Additional file 9: Table S13). As shown in Fig. 3B, we also identified the differentially expressed circRNAs in each type of cancer, and the detailed upregulated and downregulated circRNAs are listed in Additional file 10: Table S14. Interestingly, GBM (*n* = 603) showed the most differentially expressed circRNAs while GC (*n* = 123) had the least (Additional file 4: Fig. S7B). Next, we investigated whether these differentially expressed circRNAs were cancer-specific. As expected, each cancer exhibited unique patterns of differentially expressed circRNAs (Fig. 3B-C). Among the 1209 dysregulated circRNAs, 962 (79.57%) were found to be differentially expressed in only one cancer type (Fig. 3C and Additional file 10: Table S14), implying their cancer-specific roles in tumorigenesis.

In addition, 247 differentially expressed circRNAs were dysregulated in at least two cancer types (Fig. 3D and Additional file 10: Table S14). Moreover, 25 circRNAs were found to be differentially expressed in at least four types of cancers (Fig. 3E and Additional file 10: Table S14). For instance, hsa_circ_0072309 was consistently downregulated in our tested tumor samples except for GBM (Fig. 3E), highlighting its pivotal role in tumorigenesis. Of 247 circRNAs, the expression of 187 circRNAs exhibited consistent changes across different tumors (Fig. 3F and Additional file 10: Table S14), implying their common functions during tumorigenesis. For instance, hsa_circ_0077837 was significantly downregulated in CRC, ESCC, LUAD, GC and HCC (Fig. 3E and Additional file 4: Fig. S8A), indicating its universal tumor-suppressive role in cancer, which was recently proven in HCC [28]. In addition, abnormal expression of 60 circRNAs exhibited inconsistent tendencies among cancer types (Fig. 3F and Additional file 10: Table S14), suggesting their diverse roles in different cancers. For example, we found that hsa_circ_0001681 was elevated in 4 cancer types (CRC, LUAD, GC, THCA) but dramatically downregulated in GBM (Fig. 3E and Additional file 4: Fig. S8B). Consistent with our findings, hsa_circ_0001681 was previously reported to be upregulated and exert an oncogenic function in THCA [29], but to be downregulated and play a tumor-suppressive role in renal cell carcinoma [30], Therefore, such dysregulated circRNAs may exert their different functions in a cancer-type context.

### Abnormal expression of the host genes and/or RBPs contribute to circRNA dysregulation in cancer

Of note, the prevalence of differential expression profiles of circRNAs in cancer further raised the question of how these circRNAs were dysregulated. Vo et al. reported that the dysregulation of some circRNAs in prostate cancer was predominantly caused by aberrant expression of their parental genes [7]. In our study, an average of 41.84% (ranging from 33.50 to 57.81%) of differentially expressed circRNAs were explained by differentially expressed genes (Additional file 4: Fig. S9). For instance, hsa_circ_0099329 and its host gene PPFIA2 were exclusively downregulated in GBM (Additional file 4: Fig. S10A). In addition, we also observed that the alterations of some circRNAs (ranging from 42.19 to 66.50%) were inconsistent with their host genes (Additional file 4: Fig. S9), suggesting the involvement of other mechanisms in circRNA dysregulation.

Given that 66.50% of dysregulated circRNAs in GBM were derived from genes without significant changes in expression, we focused on GBM to explore whether the dysregulated circRNAs were associated with RBPs. We first examined the expression patterns of 215 RBPs involving deaminase, endonuclease, helicase and splicing factor in GBM (Additional file 11: Table S15). As shown in Fig. 3G, these RBPs such as well-characterized PTBP1 [31] and ESRP1 [32] were generally observed to be altered in GBM. Of these, PTBP1 was elevated in GBM, suggesting its potential to regulate biogenesis of some circRNAs, which wa supported by a previous report [31]. In addition, ESRP1 was decreased in GBM, implying that the downregulation of circRNAs in GBM might be affected by this RBP.

We quantified the correlations between dysregulated RBPs and dysregulated circRNAs from nondysregulated genes, and found that 174 (28.86%) circRNAs showed a strong correlation with RBPs (Pearson correlation coefficient ≥ 0.7), implying that the dysregulation of these circRNAs might be regulated by RBPs. Intriguingly, among these dysregulated RBPs, we noticed that RBFOX1, a neuron-specific splicing factor involved in neurodevelopmental and neuropsychiatric disorders [26, 33], was the most significantly downregulated RBP in GBM in our study (Fig. 3G and Additional file 4: Fig. S10B) and in the GEPIA database. Therefore, we examined the differentially expressed circRNAs associated with RBFOX1 and found that downregulation of hsa_circ_0111312 in GBM was strongly correlated with decreased RBFOX1 expression in GBM (Additional file 4: Fig. S10C), independent of expression of its host gene RASAL2 (Additional file 4: Fig. S10D). Furthermore, eCLIP data of RBFOX1 [21] confirmed the strong binding of RBFOX1 to the flanking introns of hsa_circ_0111312 (Fig. 3H), suggesting the involvement of RBFOX1 in circRNA biogenesis. Therefore, aberrant expression of RBPs in GMB can contribute to a subset of circRNA dysregulation.

### Characterization of circLIFR in cancer cells

**Fig. 4:**
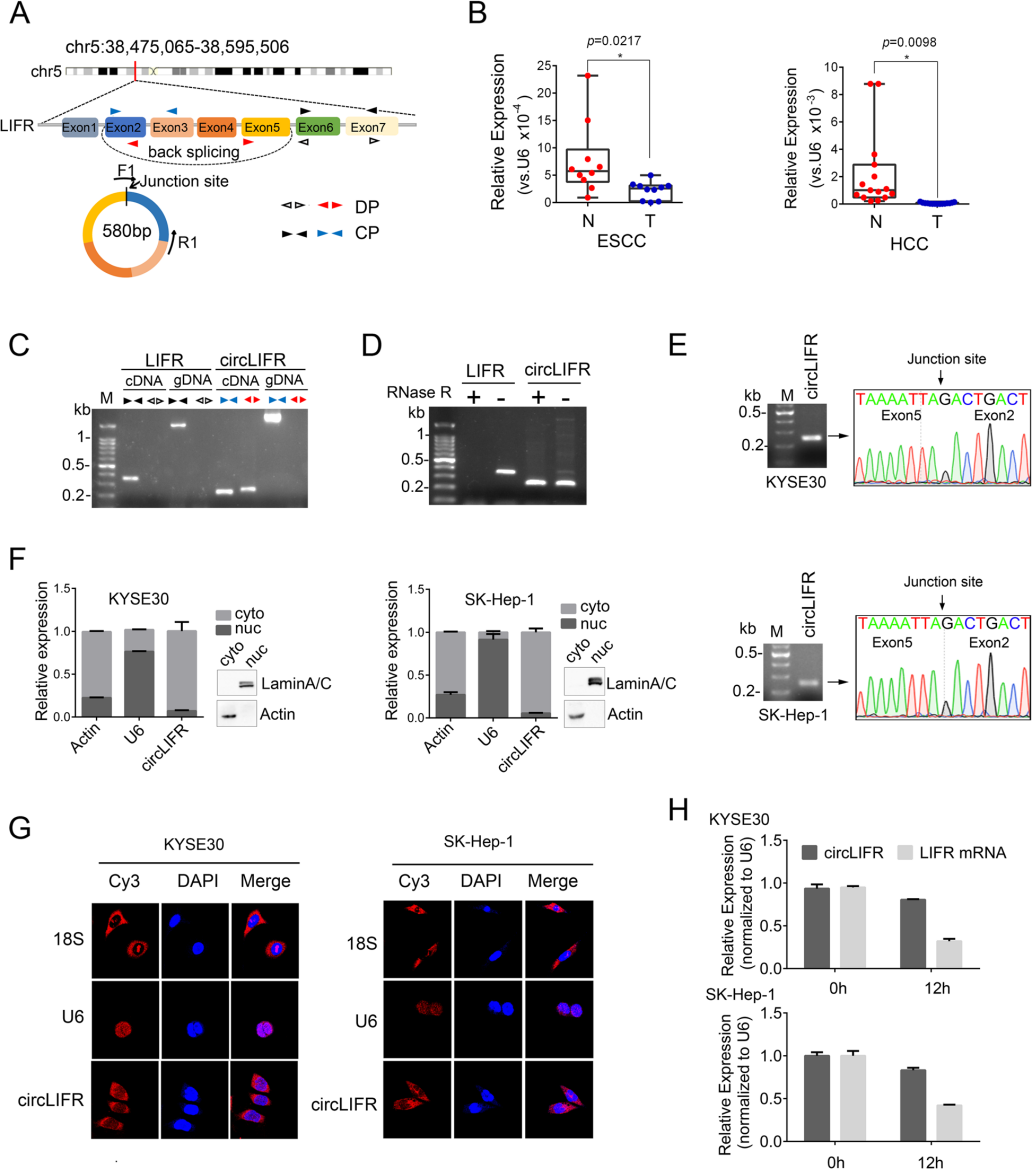
Characterization of circLIFR in solid tumors. **A** Schematic illustration showing circLIFR is derived from exons 2–5 of LIFR mRNA. The F1/R1 primers were used to measure circLIFR by RT-qPCR. DP, divergent primers; CP, convergent primers. **B** circLIFR expression measured by RT-qPCR in ESCC, HCC and their matched adjacent normal tissues, normalized to U6 RNA levels. N, normal tissues; T, tumor tissues. Data represent the mean ± S.D., and the *p-*value was determined by two-tailed paired Student’s *t*-test. **C** The presence of circLIFR was validated in KYSE150 cells by RT-PCR. Divergent primers were used to amplify circLIFR from cDNA, but not from genomic DNA. LIFR mRNA linear transcripts were used as a negative control. cDNA, complementary DNA; gDNA, genomic DNA. **D** Total RNA from KYSE150 cells with or without RNase R treatment was subjected to RT-PCR. **E** Agarose gel electrophoresis and Sanger sequencing of RT-PCR products of circLIFR in KYSE30 and SK-Hep-1 cells. **F** RT-qPCR analyses of circLIFR after cytoplasmic/nuclear fractionation of KYSE30 and SK-Hep-1 cells. β-actin mRNA and U6 RNA represent cytoplasmic and nuclear RNAs, respectively. Western blotting confirmed the efficiency of nuclear/cytoplasmic isolation. Data are shown as the mean ± SD. **G** Fluorescence in situ hybridization of circLIFR in KYSE30 and SK-Hep-1 cells. 18S and U6 RNA represent cytoplasmic and nuclear RNAs, respectively. **H** circLIFR and LIFR mRNA levels in KYSE30 and SK-Hep-1cells without or with actinomycin D treatment

According to our bioinformatics analysis, hsa_circ_0072309 exhibited high abundance in normal tissues and was downregulated in our tested tumors except for GBM. Since hsa_circ_0072309 is derived from exons 2–5 of the LIFR gene (Fig. 4A), we termed it as circLIFR. First, we designed the F1/R1 divergent primers (F1 primer crossing the junction site) to conduct RT-qPCR, and the results confirmed that circLIFR was indeed downregulated in our tested tumors, except for GBM, compared with their adjacent normal tissues (Fig. 4B and Additional file 4: Fig. S11A), implying that circLIFR could play a tumor-suppressive role in solid tumors. To further characterize circLIFR, we performed PCR with divergent (red) and convergent primers (blue) (Fig. 4A) using cDNA and genomic DNA (gDNA) as templates. As shown in Fig. 4C, the expected bands were observed using convergent primers from both cDNA and gDNA templates, but there was no band using gDNA as a template and the divergent primers, confirming that circLIFR was a circular RNA produced by back-splicing, which was further validated by RNase R digestion treatment (Fig. 4D) and Sanger sequencing in different cancer cells (Fig. 4E and Additional file 4: Fig. S11B). To detect the cellular distribution of circLIFR, we isolated the cytoplasmic and nuclear fractions of cells to measure circLIFR levels by RT-qPCR, and found that circLIFR was predominantly located in the cytoplasm (Fig. 4F and Additional file 4: Fig. S11C), which was confirmed by FISH experiments (Fig. 4G and Additional file 4: Fig. S11D). We also evaluated circLIFR stability after actinomycin D treatment and found that circLIFR was more stable than its linear transcript LIFR mRNA (Fig. 4H). Collectively, these results demonstrated that circLIFR was a bona fide circRNA, predominantly distributed in the cytoplasm and significantly downregulated in our tested tumors except for GBM.

### circLIFR inhibits cell migration in vitro and tumor metastasis in vivo

**Fig. 5:**
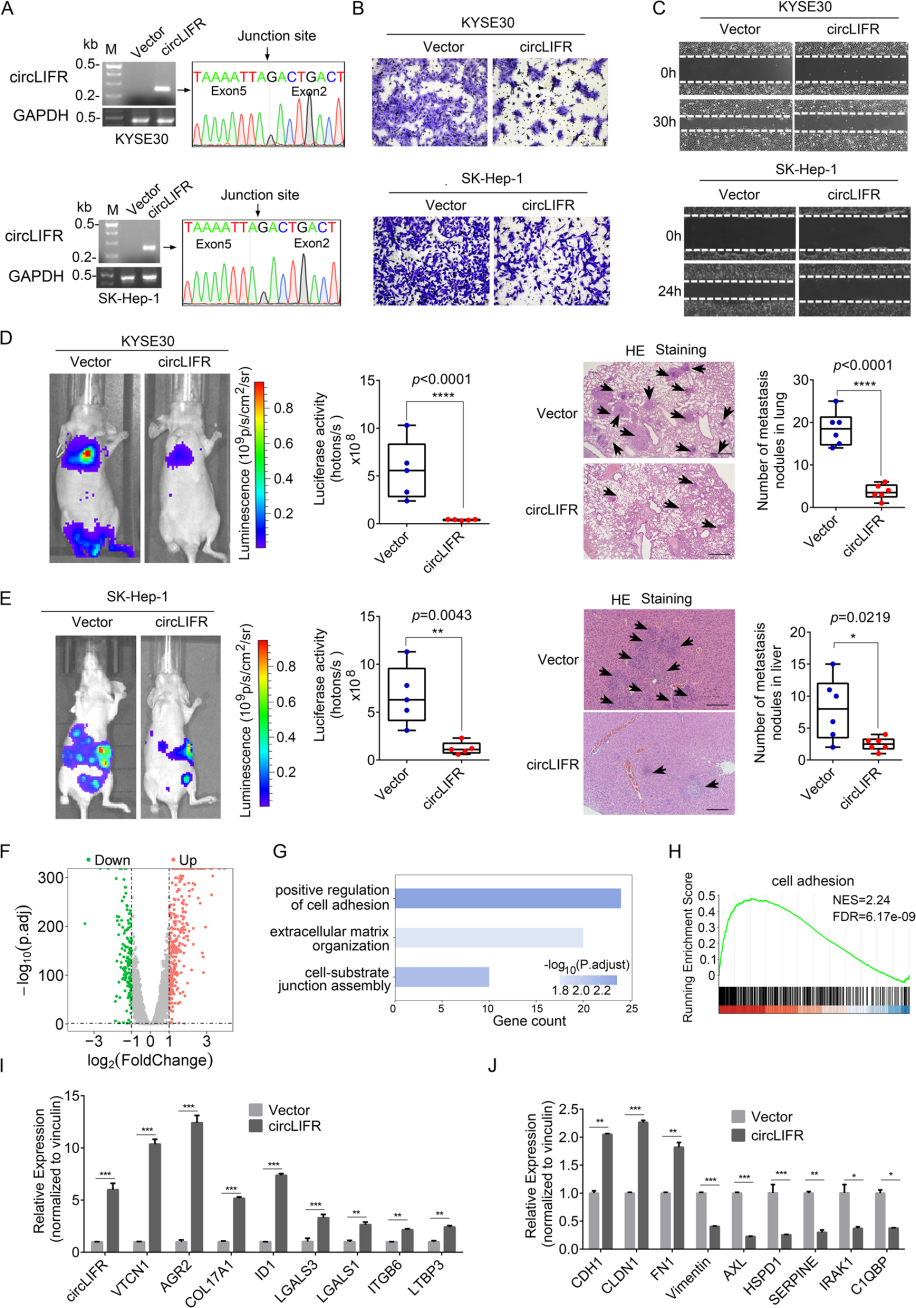
circLIFR inhibits cell migration in vitro and tumor metastasis in vivo*.* **A** Agarose gel electrophoresis and Sanger sequencing of semi-quantitative RT-PCR products demonstrated that circLIFR was correctly circularized and successfully overexpressed in KYSE30 and SK-Hep-1 cells. **B** Transwell migration assays showed that circLIFR overexpression inhibited the migratory ability of KYSE30 and SK-Hep-1 cells. **C** Wound healing assays showed that circLIFR overexpression inhibited cell migration in both KYSE30 and SK-Hep-1 cells. **D** Decreased tumor metastasis formed in the lungs of mice via tail vein injection of circLIFR-overexpressing KYSE30 cells, as indicated by representative bioluminescent images of lung metastasis at 6 weeks (left) and H&E staining of lung metastatic lesions (right) of mice for each group (*n* = 5 mice/group). **E** Decreased tumor metastasis formed in the livers of mice via spleen injection of circLIFR-overexpressing SK-Hep-1 cells, as indicated by representative bioluminescent images of liver metastasis at 8 weeks (left) and H&E staining of liver metastatic lesions (right) of mice for each group (n = 5 mice/group). **F** Volcano plot of differentially expressed genes affected by circLIFR in KYSE30 cells. **G,H** Gene Ontology (**G**) and GSEA analysis (**H**) showed that dysregulated genes upon circLIFR overexpression was involved in cell adhesion. NES, normalized enrichment score; FDR, false discovery rate. *p-*values are calculated by permutation test. **I**,**J** Quantification of metastasis-related genes by RT-qPCR in KYSE30 control cells and circLIFR-overexpressing cells. Data represented mean ± S.D.; the *p-*values were determined by a two-tailed unpaired Student’s *t*-test

circLIFR was reported to affect cellular functions in breast cancer [34], bladder cancer [35], renal carcinoma [36] and gastric cancer [37]. However, there are no reports of circLIFR in ESCC, HCC, THCA and CRC. To investigate the biological functions of circLIFR in these tumors, we first measured circLIFR levels in cancer cell lines (Additional file 4: Fig. S12A) and selected 4 cancer cells with low circLIFR expression (KYSE30 and KYSE150 esophageal cancer cells, SK-Hep-1 liver cancer cells, HCT-116 colorectal cancer cells) to construct stable cells overexpressing circLIFR. As shown in Fig. 5A and Additional file 4: Fig. S12B, circLIFR was correctly circularized and successfully expressed in these cells. Transwell migration and wound healing assays showed that overexpression of circLIFR apparently inhibited cell migration and cell motility (Fig. 5B-C and Additional file 4: Fig. S12C-D). Next, we sought to evaluate the function of circLIFR in vivo. To this end, we conducted a tail-vein injection mouse model using circLIFR-overexpressing KYSE30 cells to check its role in lung metastasis. The results demonstrated that after ectopic expression of circLIFR in esophageal cancer cells, tumor metastasis was significantly inhibited compared to that of control cells in lung metastasis models (Fig. 5D). To further evaluate the function of circLIFR, we injected circLIFR-overexpressing SK-Hep-1 cells into the spleen, and observed that circLIFR dramatically repressed tumor metastasis to mouse liver (Fig. 5E). Together, circLIFR significantly inhibited cell migration in vitro and tumor metastasis in vivo.

To explore the underlying mechanism of circLIFR, we performed RNA-seq for transcriptome analyses. As shown in Fig. 5F, ectopic expression of circLIFR affected the expression levels of 448 genes, of which 292 genes were upregulated and 156 genes were downregulated, including epithelial-mesenchymal transition (EMT) marker genes (Additional file 12: Table S16). Gene Ontology analyses indicated that these differentially expressed genes were significantly enriched in cell adhesion, extracellular matrix organization and cell-substrate junction assembly (Fig. 5G), which was further confirmed by GSEA analysis results (Fig. 5H). Finally, the expression change of a subset of metastasis-related genes affected by circLIFR was validated by RT-qPCR (Fig. 5I-J). Therefore, circLIFR inhibits tumor metastasis by regulating a subset of cell adhesion and EMT-related genes.

## Discussion

Accumulating evidence has demonstrated that circRNAs are frequently altered in cancer and play indispensable roles during cancer initiation and progression. Recently, several studies have attempted to present the landscape of circRNAs in tumor tissues and cancer cell lines, shedding light on the importance of circRNAs [6, 7, 38, 39]. However, the lack of matched normal tissues in these studies cannot depict the dysregulated circRNAs across cancer types. Therefore, we collected 122 tumor samples of seven types of malignancies (GBM, ESCC, LUAD, HCC, GC, THCA and CRC) and their matched normal tissues to conduct Ribo-zero RNA-seq, which allows us to systematically profile circRNAs in normal tissues and their matched tumors without bias.

From these samples, we identified 59,056 circRNAs, most of which were originated from exons (*n* = 59,020). Interestingly, we found that 182 circRNAs harbored a complete CDS as they originated from both the CDS and UTR, indicating their potential for translation into intact peptides. Consistent with previous studies [3, 40], we found that the majority of circRNAs were expressed at low levels. However, we identified 22 circRNAs with much higher abundance than their cognate linear transcripts (Additional file 3: Table S6), suggesting their important roles in basic cellular functions. Although a single gene locus can produce multiple circRNAs via alternative circularization, this gene shows a tendency to express a predominant circRNA isoform (Fig. 1G-H). This could be explained by our analysis that the introns flanking circularized exons of the predominant circRNA isoform are much longer and have more repetitive elements (Fig. 1I-J). In addition, we identified 30 circRNAs that were ubiquitous across our tested tissues, and 439 circRNAs were tissue-specific, indicating their general and tissue-specific cellular functions (Additional file 7: Table S10 and Additional file 8: Table S11).

Compared with normal tissues, the global abundance of circRNAs was remarkably decreased in tumors (Fig. 3A), which may be caused by inefficient back-splicing events or the dose dilution of nascent circRNAs in the rapid division of tumor cells [41, 42]. This was further supported by the fact that the total abundance of circRNAs was significantly elevated when inhibiting cell proliferation [7]. Moreover, we identified 1209 differentially expressed circRNAs in cancers (Additional file 10: Table S14), of which 962 (79.57%) were cancer-specific, while 247 were dysregulated in at least two cancer types. Of the 247 noncancer-specific circRNAs, 187 circRNAs were consistently altered in tumors, while 60 circRNAs exhibited distinct expression patterns in different cancer types, indicating that consistently altered circRNAs exert a common role in cancers, while inconsistently altered circRNAs may play distinct functions in different cancers. For instance, hsa_circ_0001681 was elevated in papillary thyroid cancer and promoted tumor growth by sponging miR-198 [29], while downregulation of circRAPGEF5 in renal cell carcinoma inhibited cancer progression by sequestering miR-27a-3p [30], supporting its diverse roles in cancers.

Given that some circRNAs originating from cancer tissues may enter the circulatory system and be stably present in sera [43, 44], cancer-specific dysregulated circRNAs could serve as biomarkers for cancer diagnosis and prognosis. For instance, the *EML4-ALK* fusion gene produces a novel F-circEA in non-small cell lung cancer (NSCLC), and the existence of F-circEA in sera makes it a promising liquid biopsy biomarker for monitoring the *EML4-ALK* fusion gene [45]. Moreover, circRNAs driving tumor progression could be therapeutic targets. For example, circPRKCI was demonstrated to be a proto-oncogenic circular RNA in lung adenocarcinoma tissues, and the intratumor injection of cholesterol-conjugated siRNA specifically targeting circPRKCI inhibited tumor growth in a patient-derived lung adenocarcinoma xenograft model [46]. Therefore, elucidation of the biological functions and clinical relevance of such dysregulated circRNAs is helpful to identify new diagnostic biomarkers and therapeutic targets.

Our study provided abundant dysregulated circRNAs to be experimentally validated for their roles in tumorigenesis. Here we focused on hsa_circ_0072309 (circLIFR) because of its consistent decrease in LUAD, ESCC, CRC, GC, HCC and THCA. First, we confirmed the downregulation of circLIFR in these six types of cancers by RT-qPCR, supporting the robustness of our pipeline for transcriptome analyses. Combined with recent findings about circLIFR in breast cancer [34], renal carcinoma [36] and bladder cancer [35], downregulation of circLIFR seems to be general in cancers, suggesting its important tumor-suppressive roles in cancer pathology. Indeed, circLIFR was reported to repress the progression of breast cancer [34], renal carcinoma [36] and GC [37]. In esophageal cancer and liver cancer models, we verified that circLIFR inhibited cell migration in vitro and tumor metastasis in vivo. Therefore, circLIFR plays a pivotal role during tumorigenesis. To explore the effects of circLIFR on gene expression, we performed RNA-seq on circLIFR-overexpressing KYSE30 cells and revealed that circLIFR significantly altered the expression patterns of metastasis-related genes (such as cell adhesion molecules). Mechanistically, circLIFR was reported to act as a sponge of miR-492 in breast cancer [34] and miR-100 in renal carcinoma [36] to inhibit tumor progression. In bladder cancer, circLIFR interacted with the MSH2 protein to positively modulate cisplatin-sensitivity through the MutSα/ATM-p73 axis [35]. However, the detailed underlying mechanism of circLIFR in esophageal cancer is still vague and needs further investigation.

In addition, we characterized some tissue-specific circRNAs in our tested normal tissues and cancer-specific dysregulation of circRNAs in tumors. Given that circRNA biogenesis is regulated by *cis*-acting elements and *trans*-acting proteins, we analyzed the factors to determine circRNA expression. As shown in Fig. 2C and Additional file 4: Fig. S5A, we found that the tissue-specific expression of the host genes contributes to 30.75% of tissue-specific circRNAs, while aberrant expression of the host gene contributes to approximately 41.84% of dysregulated circRNAs, indicating that the expression levels of host genes are an important factor for circRNA expression. Moreover, we identified that 35.31% of tissue-specific circRNAs and 28.86% of dysregulated circRNAs in GBM were highly correlated with RBPs, which was further supported by published eCLIP-seq data (Fig. 2I and Fig. 3H), demonstrating pivotal roles in RBPs in circRNA biogenesis. Because we found a strong association between dysregulated RBPs (such as PTBP1, RBFOX1 and ESRP1) and the expression of a subset of circRNAs in GBM, it is important to dissect how these RBPs affect circRNA biogenesis in the future.

## Conclusions

In this study, we identified the expression landscape of circRNAs in paired normal-tumor clinical samples from seven types of malignancies. The dysregulated circRNAs exhibited cancer-specific expression or shared common expression signatures across cancers, which could be regulated by the expression of host genes and RBPs. Finally, we experimentally validated that circLIFR was downregulated and played a tumor-suppressive role in tumors. Collectively, this study provides the comprehensive profiles of differentially-expressed circRNAs in cancers and highlights the important roles of circRNAs in cancer biology.

## Supplementary Information

**Additional file 1: Table S1.** Clinical characteristics of the patients enrolled in this study.

**Additional file 2: Table S2.** The information of 215 RBPs that associated with circRNA biogenesis.

**Additional file 3: Table S3.** Information of primers and probes used in this study. **Table S4.** The distribution of putative spliced length of exonic circRNAs. **Table S5.** Exon numbers of identified exonic circRNAs. **Table S6.** The circRNAs expressed higher than its host genes. **Table S7.** The number of circRNAs produced from one gene. **Table S12.** Information of tissue-specific RBPs.

**Additional file 4: Figure S1.** Characterization of protein-coding gene profiles in normal tissues. **Figure S2.** Bioinformatics validation of tissue-specifically expressed genes using the GTEx dataset. Gene expression was measured in transcripts per million (TPM). **Figure S3.** The genes with tissue-specific expression patterns were generally enriched in the physiological processes (A) and signaling pathways (B) that were specific to the corresponding tissue. **Figure S4.** The tissue-specific expression patterns of circRNAs were confirmed by the circAtlas database. **Figure S5.** The tissue specificity of a subset of circRNAs was governed by their cognate genes. Examples of tissue-specific circRNAs corresponding to their host gene expression. B-D Bioinformatics validation of brain-specifically expressed SLAIN1 using FANTOM5 (B), GTEx (C) and HPA (D) datasets. **Figure S6.** Brain-specifically expressed hsa_circ_0073539 was regulated by the brain-specific splicing factor QKI. A Brain-specific expression of hsa_circ_0111312 was independent of its host gene (MAN2A1) expression. B The Pearson correlation coefficients between brain-specific hsa_circ_0073539 and the splicing factor QKI. **Figure S7.** The distribution of uniquely mapped reads (A) and the number of identified upregulated and downregulated circRNAs (B) in each cancer sample. The *p*-value was calculated using the Wilcoxon rank-sum test, ns denotes no significance, * denotes *p* < 0.05, ** denotes *p* < 0.01, *** denotes *p* < 0.001. **Figure S8.** Expression patterns of dysregulated circRNAs in cancer. A,B The expression profiles of hsa_circ_0077837 (A) and hsa_circ_0001681 (B) in different cancers. The *p-*value was calculated using the Wilcoxon rank-sum test, ns denotes no significance, * denotes *p* < 0.05, ** denotes *p* < 0.01, *** denotes *p* < 0.001. **Figure S9.** Comparison of expression changes between differentially expressed circRNAs and their parental genes among different cancers. Based on circRNA fold-change versus its linear transcript fold-change, circRNAs were divided into four groups. **Figure S10.** Aberrant expression of circRNAs in cancers may be regulated by the host genes or RBPs. A Expression of hsa_circ_0099329 and its host gene PPFIA2 in tested cancers. B The splicing factor RBFOX1 was exclusively downregulated in GBM. C The Pearson correlation coefficients between RBFOX1 and hsa_circ_0111312 in GBM. The *p-*value was calculated using the Wilcoxon rank-sum test, ns denotes no significance, * denotes *p* < 0.05, ** denotes *p* < 0.01, *** denotes *p* < 0.001. D Expression of hsa_circ_0111312 and its host gene RASAL2 in tested cancers. **Figure S11.** circLIFR expression in solid tumors and cells. A Quantification of circLIFR by RT-qPCR in solid tumors (LUAD, CRC, GC, THCA, GBM) and their matched adjacent normal tissues, and circLIFR expression was normalized to U6 mRNA levels. N, normal tissues; T, tumor tissues. B Identification of the circularization site of circLIFR in KYSE150 and HCT-116 cells by RT-PCR and Sanger sequencing. C,D Cellular distribution of circLIFR in KYSE150 and HCT-116 cells, determined by cell nucleus/cytoplasm fractionation and RT-qPCR analyses (C) and fluorescence in situ hybridization (D). β-actin mRNA and U6 RNA represent cytoplasmic and nuclear RNAs, respectively. Western blotting confirmed the efficiency of nuclear/cytoplasmic isolation. Data are shown as the mean ± SD. **Figure S12.** Effects of circLIFR on cell migration and motility in KYSE150 and HCT-116 cells.

**Additional file 5: Table S8.** The expression profiles of 1590 protein-coding genes that ubiquitously expressed in normal tissues.

**Additional file 6: Table S9.** The expression profiles of 1765 tissue-specific protein-coding genes.

**Additional file 7: Table S10.** The expression profiles of 30 circRNAs that ubiquitously expressed in normal tissues.

**Additional file 8: Table S11.** The expression profiles of 439 tissue-specific circRNAs.

**Additional file 9: Table S13.** The detailed mapping statistics of samples used in this study.

**Additional file 10: Table S14.** The expression profiles of 1209 differentially expressed circRNAs identified in this study.

**Additional file 11: Table S15.** The differential expression profiles of 215 RBPs that associated with circRNA biogenesis in GBM.

**Additional file 12: Table S16.** The differentially expressed protein-coding genes identified in circLIFR-overexpressing KYSE30 cells.

## Data Availability

All data and materials obtained and/or analyzed in this study were available from the corresponding authors upon a reasonable request.

## Abbreviations

circRNA: Circular RNA
RNA-seq: Transcriptome sequencing
EcRNAs: Exonic circRNAs
EIciRNAs: Exon-intron circRNAs
ciRNAs: Intronic circRNAs
miRNA: microrna
RBP: RNA-binding protein
SMO: Smoothened, frizzled class receptor
GBM: Glioblastoma
IGF2BP3: Insulin-like growth factor 2 mRNA binding protein 3
CCLE: Cancer Cell Line Encyclopedia
rRNA: Ribosomal RNA
ESCC: Esophageal squamous cell carcinoma
LUAD: Lung adenocarcinoma
THCA: Thyroid cancer
CRC: Colorectal cancer
GC: Gastric cancer
HCC: Hepatocellular carcinoma
LIFR: Leukemia inhibitory factor receptor alpha
RT-qPCR: Quantitative real-time PCR
PE150: Paired-end 150 bp
RPM: Reads per million mapped reads
FPKM: Fragments per kilobase per million
TSI: Tissue specificity index
GO: Gene Ontology
KEGG: Kyoto Encyclopedia of Genes and Genomes
GSEA: Gene Set Enrichment Analysis
eCLIP-seq: Enhanced UV crosslinking and immunoprecipitation followed by sequencing
RBFOX1: RNA binding protein, fox-1 homolog (*C. elegans*) 1
QKI: QKI, KH domain containing
RNA binding: RT-PCR: reverse transcription PCR
FBS: Fetal bovine serum
FISH: Fluorescence in situ hybridization
DAPI: 4′,6-diamidino-2-phenylindole
CDS: Coding sequence
UTR: Untranslated region
HIPK3: Homeodomain interacting protein kinase 3
GTEx: Genotype-Tissue Expression
SLAIN1: SLAIN motif family, member 1
PPFIA2: PTPRF interacting protein alpha 2
PTBP1: Polypyrimidine tract binding protein 1
ESRP1: Epithelial splicing regulatory protein 1
GEPIA: Gene Expression Profiling Interactive Analysis
cDNA: Complementary DNA
gDNA: Genomic DNA
EMT: Epithelial-mesenchymal transition
RAPGEF5: Rap guanine nucleotide exchange factor 5
EML4: Echinoderm microtubule associated protein like 4
ALK: Anaplastic lymphoma receptor tyrosine kinase
NSCLC: Non-small cell lung cancer
PRKCI: Protein kinase C iota
