# Supplementary material for: Characterization of distinct circular RNA signatures in solid tumors
Source: Mol Cancer. 2022 Mar 2;21:63. doi: 10.1186/s12943-022-01546-4 (PMC8889743; doi:10.1186/s12943-022-01546-4)
Supplement: Supplementary file 3 — Additional file 3: Table S3. Information of primers and probes used in this study. Table S4. The distribution of putative spliced length of exonic circRNAs. Table S5. Exon numbers of identified exonic circRNAs. Table S6. The circRNAs expressed higher than its host genes. Table S7. The number of circRNAs produced from one gene. Table S12. Information of tissue-specific RBPs. [file 12943_2022_1546_MOESM3_ESM.pdf]

Supplementary tables

Table S3. Information of primers and probes used in this study.

Table S4. The distribution of putative spliced length of exonic circRNAs.

Table S5. Exon numbers of identified exonic circRNAs.

Table S6. The circRNAs expressed higher than its host genes.

Table S7. The number of circRNAs produced from one gene.

Table S12. Information of tissue-specific RBPs.

Table S3. Information of primers and probes used in this study

Name	Sequence (5’-3’)	Usage	Target name
F1	GCTCGTAAAATTAGACTGACTGCATTG	RT-qPCR	circLIFR
R1	ATGAGGAGCCCCCTTTTTCTGG		
F2	ATTTGTCTGCTGATTTCTCAACCTC	divergent primer for PCR	
R2	TAAATGTTGATAACAGCCACTGGA		
F3	GCATTGCACAGATGATGGATATT	convergent primer for PCR	
R3	TGTTCCAGAGGGTGCTTTC		
F4	TTCTGTTTCTGCAAGTAGTGGA	divergent primer for PCR	LIFR
R4	GTATCTTTGCCATTCAGAGTTGTG		
F5	CACAACTCTGAATGGCAAAGATAC	convergent primer for PCR	
R5	TCCACTACTTGCAGAAACAGAA		
F6	ACATCATCCCTGCCTCTACT	PCR	GAPDH
R6	CTCTCTTCCTCTTGTGCTCTTG		
F7	CGCTTCGGCAGCACATATAC	RT-qPCR	U6
R7	AGGGGCCATGCTAATCTTCT		
F8	AGGCCAACCGCGAGAAGATG	RT-qPCR	Actin
R8	GCCAGAGGCGTACAGGGATA		
F9	TTCAGCGATACCACAACTCC	RT-qPCR	Vinculin
R9	AGTTAGCTGCCCTCTCATCA		
F10	ATTTTTTTTATTTTATGCAGGACTGACTG CATTGCACAGAT	Cloning	circLIFR
R10	TTGGAATTTTGAATACTTACTAATTTTACG AGCTCCATACTCTCTTTACG		
probe1	GCAATGCAGTCAGTCTAATTTTACGAGCT	FISH probe	circLIFR
probe2	GCTAATCTTCTCTGTATCGTTCCAATTTT AGTATATGTGCTGCCG	FISH probe	U6
probe3	CATGGCTTAATCTTTGAGAC	FISH probe	18S
F11	TGCTGGGACTTCCTATGTG	RT-qPCR	FN1
R11	CCTCGATTATCCTTCTTGC		
F12	CCACCAAAGTCACGCTGAAT	RT-qPCR	CDH1
R12	CCAGGAGAGGAGTTGGGAAA		
F13	CCGTTGGCATGAAGTGTATGA	RT-qPCR	CLDN1
R13	GCCAGACCTGCAAGAAGAAATA		
F14	TGTTTCCAAGCCTGACCTCA	RT-qPCR	Vimentin
R14	CGGTACTCAGTGGACTCCTG		
F15	TAGTGGGTGGGATTGAGGTATG	RT-qPCR	AGR
R15	CTCCATGGCAACTCTGGATTTC		
F16	ATTCAGAGGCATCGTTGGAC	RT-qPCR	COL17A1
R16	TAAGACGAGAGGTCCTCCAC		

F17<br>R17	CAAGCACAGCCAGTCCA<br>TCCTAGCAGTACATACCACCA	RT-qPCR	AXL
F18<br>R18	CCTCAACGGCGAGATCAG<br>ACCCACAGAGCACGTAATTC	RT-qPCR	ID1
F19<br>R19	CAGCCAACGAGCGGAAA<br>CATCCTTGAGGGTTTGGGTT	RT-qPCR	LGALS3
F20<br>R20	CCTGAATCTCAAACCTGGAGAG<br>CACAGGTTGTTGCTGTCTTTG	RT-qPCR	LGALS1
F21<br>R21	GAAGAGATGGAGCGTGCC<br>GCACATGCCGTCCTCTC	RT-qPCR	LTBP3
F22<br>R22	TATGACCCAGGTGTACGAGAG<br>AGTGCTGGACACGTAGGA	RT-qPCR	IRAK1
F23<br>R23	GCGAGTCTGAATGGAAGGATAC<br>TCGGCAAGGAAATCCATTAGG	RT-qPCR	C1QBP
F24<br>R24	GACACTCCATCACAGTCACTAC<br>ACCTTCCTTCAGCCATTGTATC	RT-qPCR	VTCN1
F25<br>R25	CGCAGGGTGTGCAGATT<br>GCGAGTGAGGGACAGAGT	RT-qPCR	HSPD1
F26<br>R26	CACATCAGCACCAAGACCATAG<br>TTCAGCGGCTCCTTCAAATC	RT-qPCR	SERPIN<br>E2
F27<br>R27	TGCTTATTGGACCTCAGTGTG<br>CTACTTGGGAGACAGGGTTTTC	RT-qPCR	ITGB6

Sequences that spanning junction site within the qPCR primers are marked in grey.

Table S4. The distribution of putative spliced length of exonic circRNAs

Putative spliced length of circRNA	CircRNA number	Percentage (%)
0~200	2,152	3.65
200~400	16,914	28.66
400~600	12,942	21.93
600~800	8,484	14.37
800~1000	5,516	9.34
1000~1200	3,646	6.18
1200~1400	2,444	4.14
1400~1600	1,736	2.94
1600~1800	1,253	2.12
1800~2000	960	1.63
>2000	2,973	5.04

Table S5. Exon numbers of identified exonic circRNAs

Exon number of exonic circRNA	CircRNA number	Percentage (%)
1	1,952	3.31
2	11,406	19.33
3	10,756	18.22
4	8,580	14.54
5	6,490	11.00
6	4,867	8.25
7	3,564	6.04
8	2,671	4.52
9	2,018	3.42
10	1,443	2.44
>10	5,273	8.93

Table S6. The circRNAs expressed higher than its host genes

circRNA	Host gene	Average junction ratio of circRNA	Average FPKM expression of gene
hsa_circ_0000471	N4BP2L2	0.7824974	46.558716
hsa_circ_0099634	RMST	0.9566105	83.866626
hsa_circ_0000284	HIPK3	0.6274854	33.628894
hsa_circ_0006374	ARCN1	0.9990338	16.093043
hsa_circ_0007383	ARCN1	0.9987578	16.093043
hsa_circ_0005589	ARCN1	0.9937304	16.093043
hsa_circ_0000835	MIB1	0.8985182	14.372139
hsa_circ_0007817	HUWE1	0.9958366	21.997406
hsa_circ_0006192	ZHX3	0.7328196	14.649046
hsa_circ_0003910	SUMF1	0.9457619	14.209606
hsa_circ_0005232	SLC8A1	0.7920198	15.600111
hsa_circ_0000994	SLC8A1	0.5780236	15.600111
hsa_circ_0008260	RGPD5	0.6850834	11.916058
hsa_circ_0006139	RGPD6	0.9840918	11.119681
hsa_circ_0007951	ABI2	0.7374374	16.954799
hsa_circ_0002316	OMA1	0.8840141	14.269048
hsa_circ_0008433	PDE4B	0.8253544	16.107127
hsa_circ_0006091	RGS12	0.5231434	10.879712
hsa_circ_0126176	PCDH7	0.9815851	12.986978
hsa_circ_0001451	FBXW7	0.7319191	16.447917
hsa_circ_0002210	ZNF706	0.5163743	25.501214
hsa_circ_0136839	ERICH1	0.9365828	10.706035

Table S7. The number of circRNAs produced from one gene

CircRNA number produced from one gene	Gene number	Percentage (%)
1	2,279	26.46
2	1,274	14.79
3	850	9.87
4	634	7.36
5	527	6.12
6	378	4.39
7	339	3.93
8	275	3.19
9	241	2.80
10	195	2.26
>10	1,622	18.83

Table S12. Information of tissue-specific RBPs

RBP	Gene ID	Type	Tissue	Tissue specificity index (TSI)
ADARB2	ENSG00000185736.11	Deaminase	Brain	0.953734
CELF3	ENSG00000159409.10	RNA splicing	Brain	0.991312
KHDRBS3	ENSG00000131773.9	RNA splicing	Brain	0.906729
NOVA1	ENSG00000139910.15	RNA splicing	Brain	0.903156
QKI	ENSG00000112531.12	RNA splicing	Brain	0.863486
RBFOX1	ENSG00000078328.15	RNA splicing	Brain	0.997668
RPP21	ENSG00000241370.1	Endonuclease	Esophagus	0.926018
SRSF12	ENSG00000154548.8	RNA splicing	Brain	0.899597
