# Supplementary material for: Characterization of distinct circular RNA signatures in solid tumors
Source: Mol Cancer. 2022 Mar 2;21:63. doi: 10.1186/s12943-022-01546-4 (PMC8889743; doi:10.1186/s12943-022-01546-4)
Supplement: Supplementary file 4 — Additional file 4: Figure S1. Characterization of protein-coding gene profiles in normal tissues. Figure S2. Bioinformatics validation of tissue-specifically expressed genes using the GTEx dataset. Gene expression was measured in transcripts per million (TPM). Figure S3. The genes with tissue-specific expression patterns were generally enriched in the physiological processes (A) and signaling pathways (B) that were specific to the corresponding tissue. Figure S4. The tissue-specific expression patterns of circRNAs were confirmed by the circAtlas database. Figure S5. The tissue specificity of a subset of circRNAs was governed by their cognate genes. Examples of tissue-specific circRNAs corresponding to their host gene expression. B-D Bioinformatics validation of brain-specifically expressed SLAIN1 using FANTOM5 (B), GTEx (C) and HPA (D) datasets. Figure S6. Brain-specifically expressed hsa_circ_0073539 was regulated by the brain-specific splicing factor QKI. A Brain-specific expression of hsa_circ_0111312 was independent of its host gene (MAN2A1) expression. B The Pearson correlation coefficients between brain-specific hsa_circ_0073539 and the splicing factor QKI. Figure S7. The distribution of uniquely mapped reads (A) and the number of identified upregulated and downregulated circRNAs (B) in each cancer sample. The p-value was calculated using the Wilcoxon rank-sum test, ns denotes no significance, * denotes p < 0.05, ** denotes p < 0.01, *** denotes p < 0.001. Figure S8. Expression patterns of dysregulated circRNAs in cancer. A,B The expression profiles of hsa_circ_0077837 (A) and hsa_circ_0001681 (B) in different cancers. The p-value was calculated using the Wilcoxon rank-sum test, ns denotes no significance, * denotes p < 0.05, ** denotes p < 0.01, *** denotes p < 0.001. Figure S9. Comparison of expression changes between differentially expressed circRNAs and their parental genes among different cancers. Based on circRNA fold-change versus its linear transcript [file 12943_2022_1546_MOESM4_ESM.pdf]

Supplementary figures

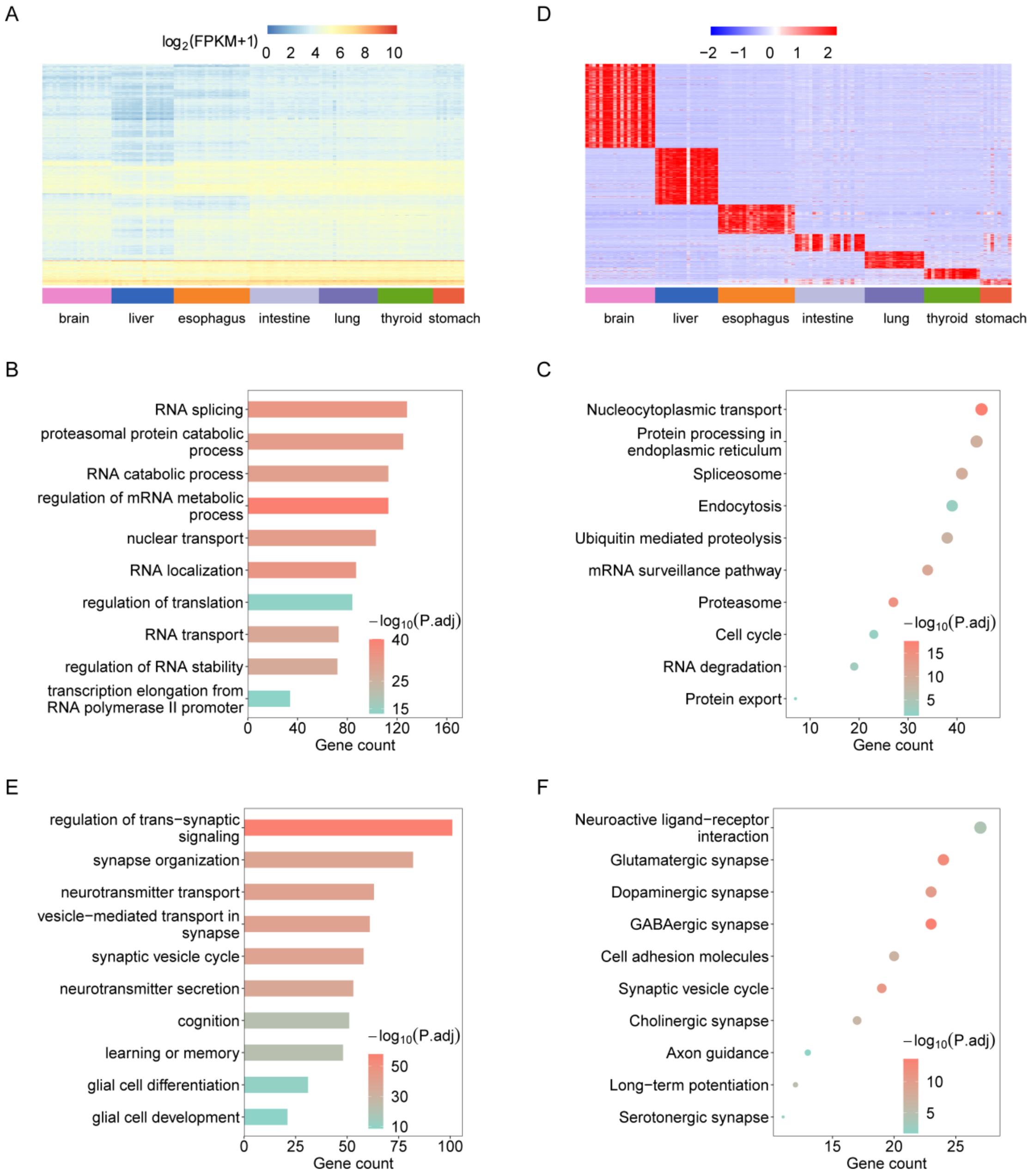

Fig S1. Characterization of protein-coding gene profiles in normal tissues. **A** Heatmap of ubiquitously expressed protein-coding genes. **B**,**C** Enriched biological processes (**B**) and signaling pathways (**C**) of the ubiquitously expressed genes. **D** Heatmap of tissue-specifically expressed protein-coding genes. **E**,**F** Enriched biological processes (**E**) and signaling pathways (**F**) of brain-specifically expressed genes.

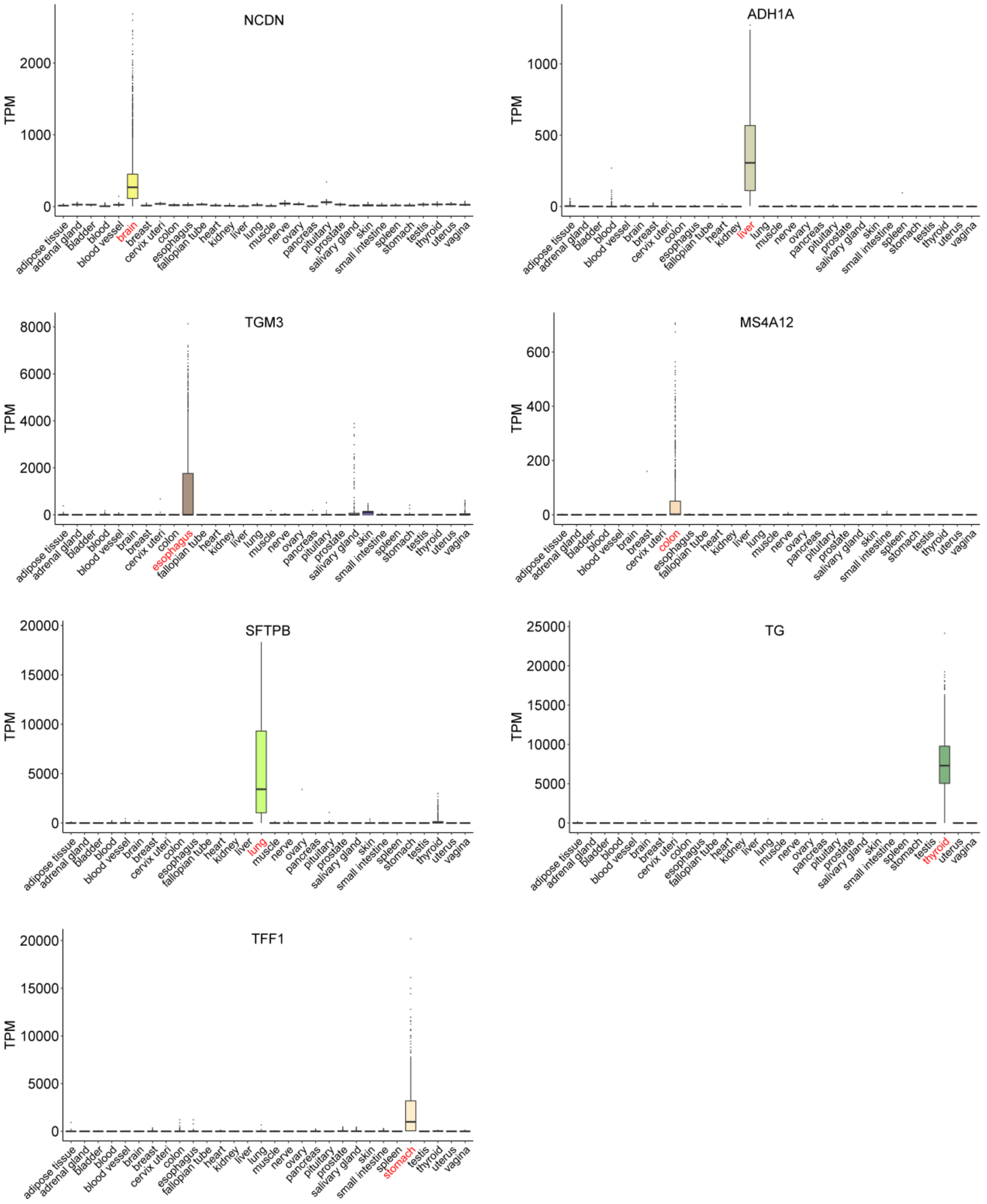

Fig S2. Bioinformatics validation of tissue-specifically expressed genes using the GTEx dataset. Gene expression was measured in transcripts per million (TPM).

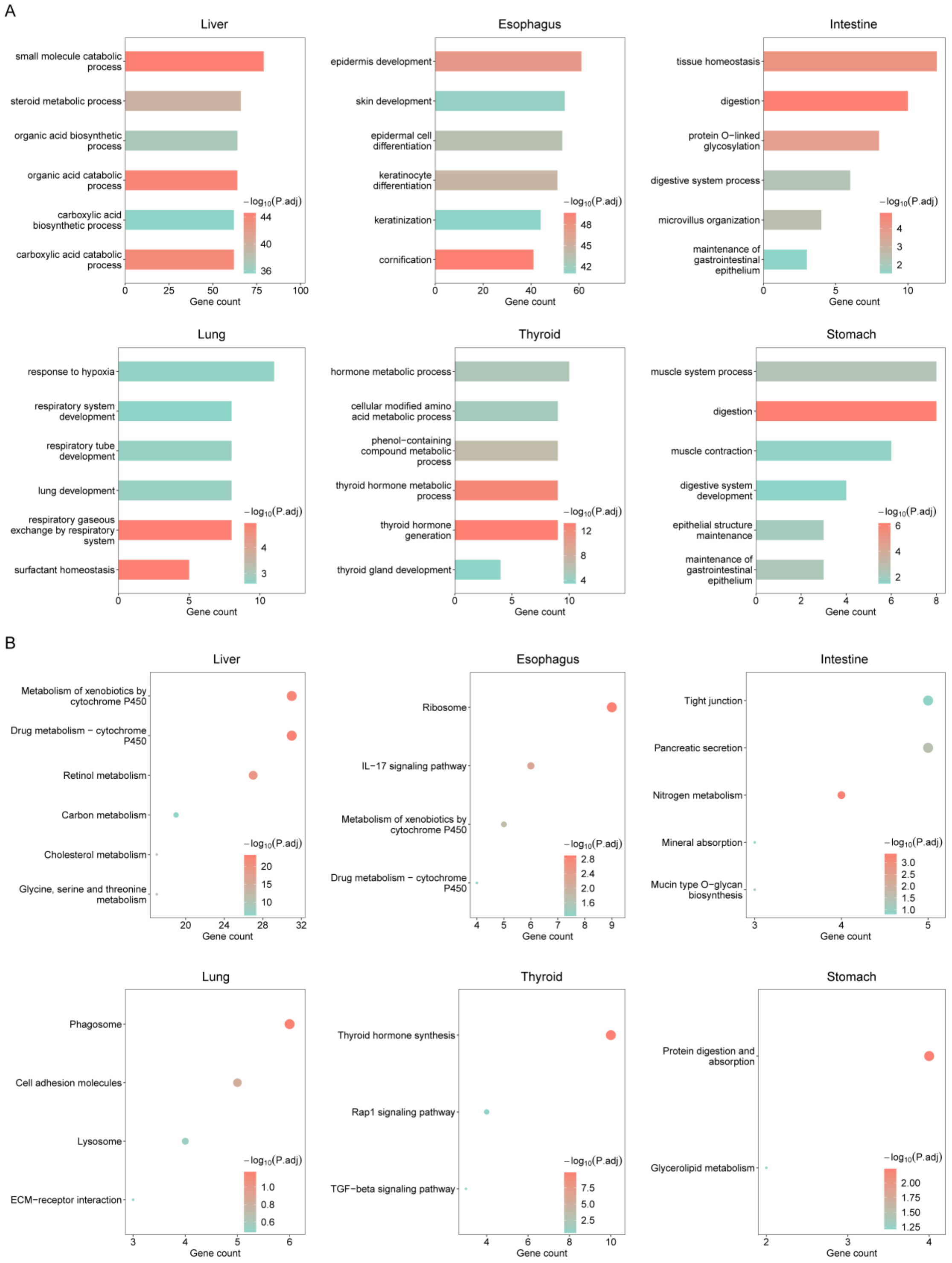

Fig S3. The genes with tissue-specific expression patterns were generally enriched in the physiological processes (**A**) and signaling pathways (**B**) that were specific to the corresponding tissue.

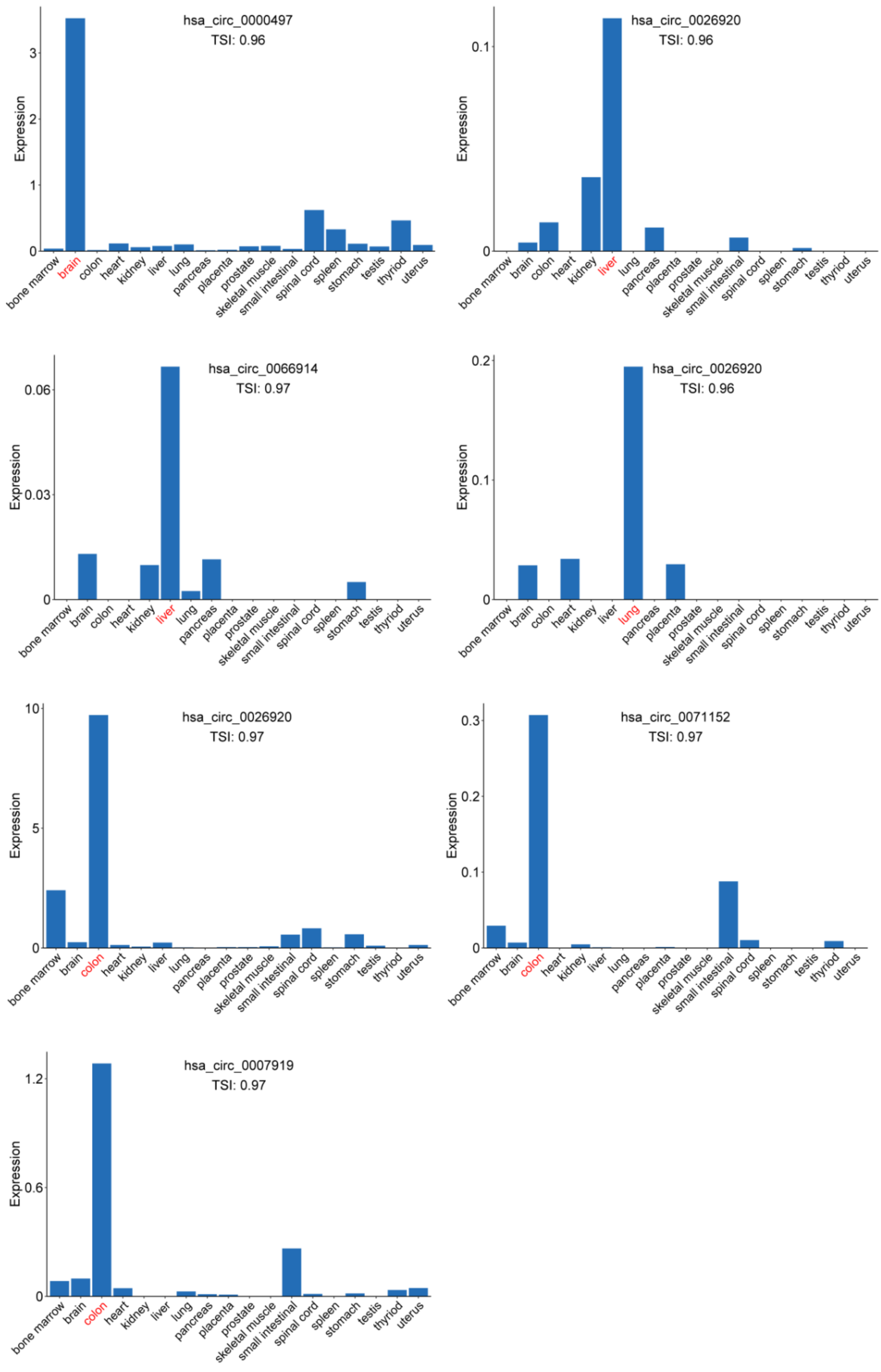

Fig S4. The tissue-specific expression patterns of circRNAs were confirmed by the circAtlas database.

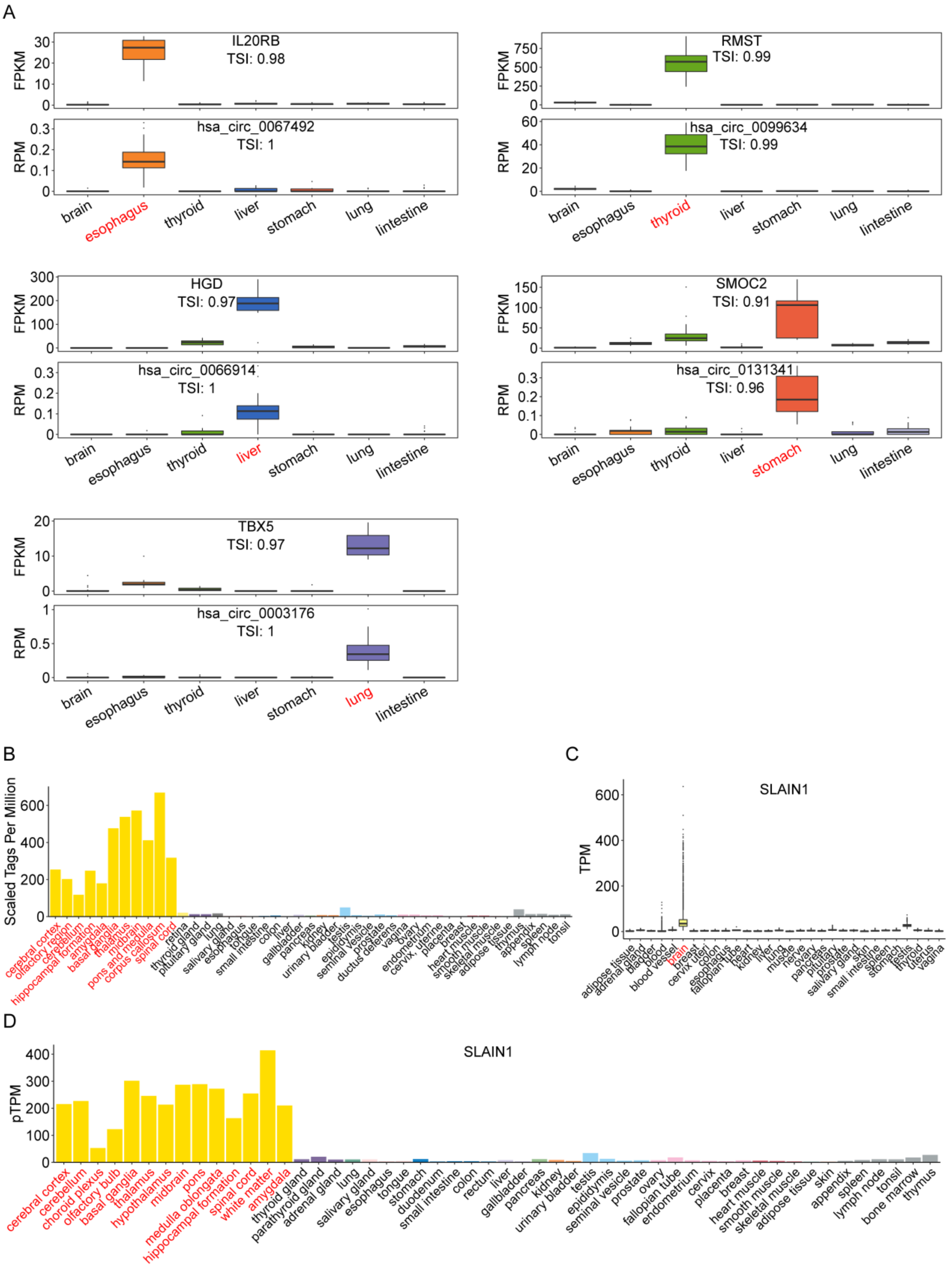

Fig S5. The tissue specificity of a subset of circRNAs was governed by their cognate genes. **A** Examples of tissue-specific circRNAs corresponding to their host gene expression. **B**-**D** Bioinformatics validation of brain-specifically expressed SLAIN1 using FANTOM5 (**B**), GTEx (**C**) and HPA (**D**) datasets.

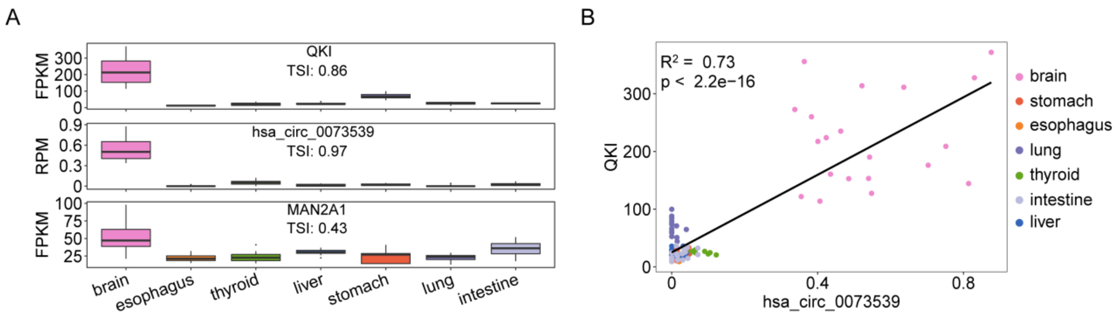

Fig S6. Brain-specifically expressed hsa_circ_0073539 was regulated by the brain-specific splicing factor QKI. **A** Brain-specific expression of hsa_circ_0111312 was independent of its host gene (MAN2A1) expression. **B** The Pearson correlation coefficients between brain-specific hsa_circ_0073539 and the splicing factor QKI.

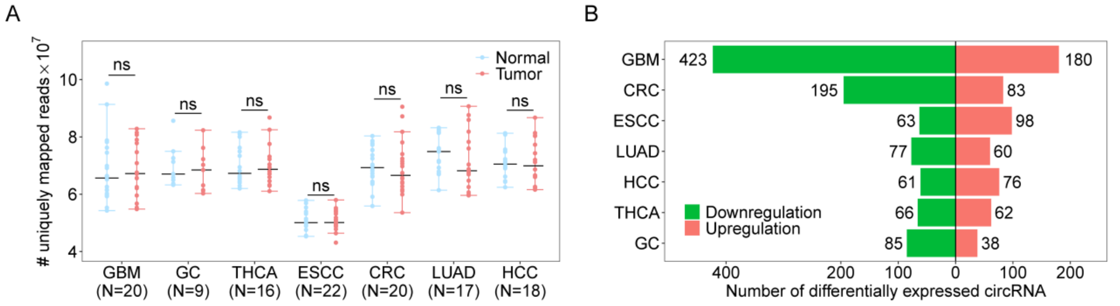

Fig S7. The distribution of uniquely mapped reads (**A**) and the number of identified upregulated and downregulated circRNAs (**B**) in each cancer sample. The *p*-value was calculated using the Wilcoxon rank-sum test, ns denotes no significance, * denotes $p < 0.05$, ** denotes $p < 0.01$, *** denotes $p < 0.001$.

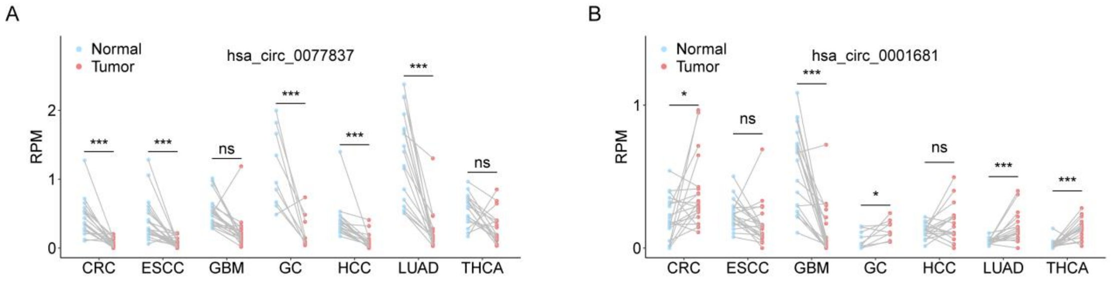

Fig S8. Expression patterns of dysregulated circRNAs in cancer. **A**,**B** The expression profiles of hsa_circ_0077837 (**A**) and hsa_circ_0001681 (**B**) in different cancers. The *p*-value was calculated using the Wilcoxon rank-sum test, ns denotes no significance, * denotes $p < 0.05$, ** denotes $p < 0.01$, *** denotes $p < 0.001$.

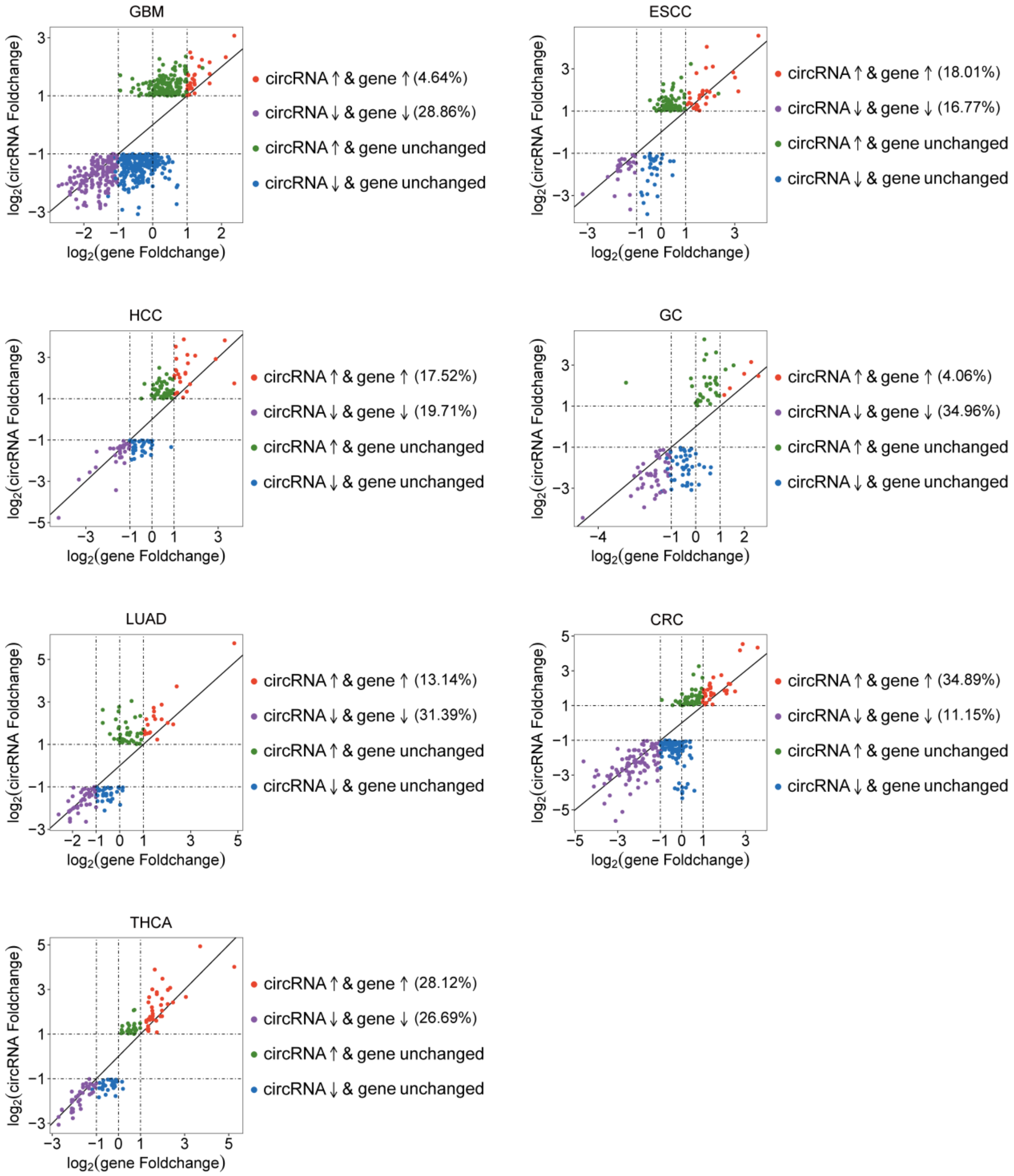

Fig S9. Comparison of expression changes between differentially expressed circRNAs and their parental genes among different cancers. Based on circRNA fold-change versus its linear transcript fold-change, circRNAs were divided into four groups.

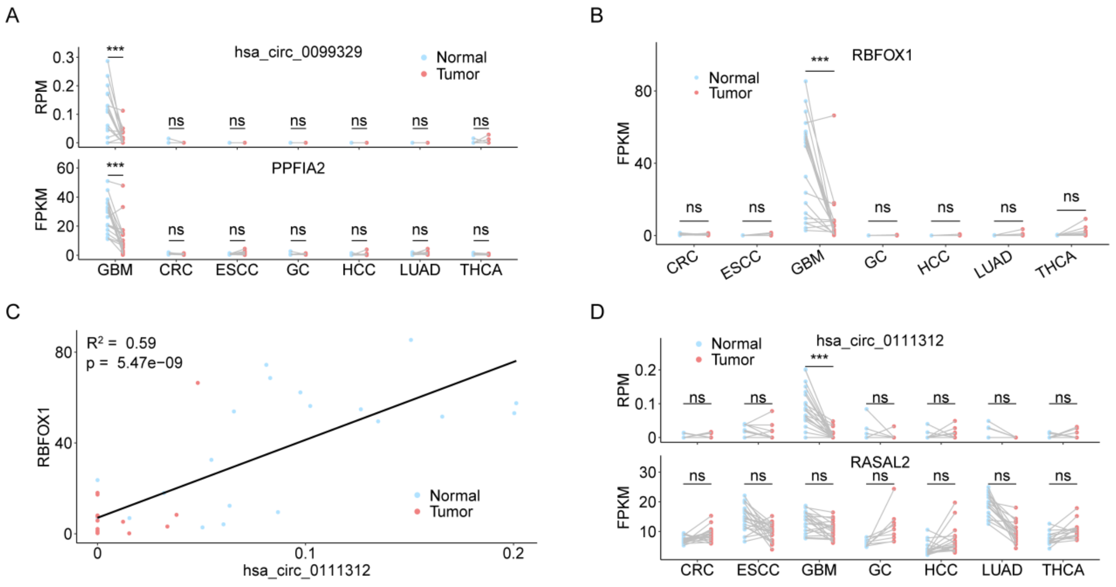

Fig S10. Aberrant expression of circRNAs in cancers may be regulated by the host genes or RBPs. **A** Expression of hsa_circ_0099329 and its host gene PPFIA2 in tested cancers. **B** The splicing factor RBFOX1 was exclusively downregulated in GBM. **C** The Pearson correlation coefficients between RBFOX1 and hsa_circ_0111312 in GBM. The *p*-value was calculated using the Wilcoxon rank-sum test, ns denotes no significance, * denotes $p < 0.05$, ** denotes $p < 0.01$, *** denotes $p < 0.001$. **D** Expression of hsa_circ_0111312 and its host gene RASAL2 in tested cancers.

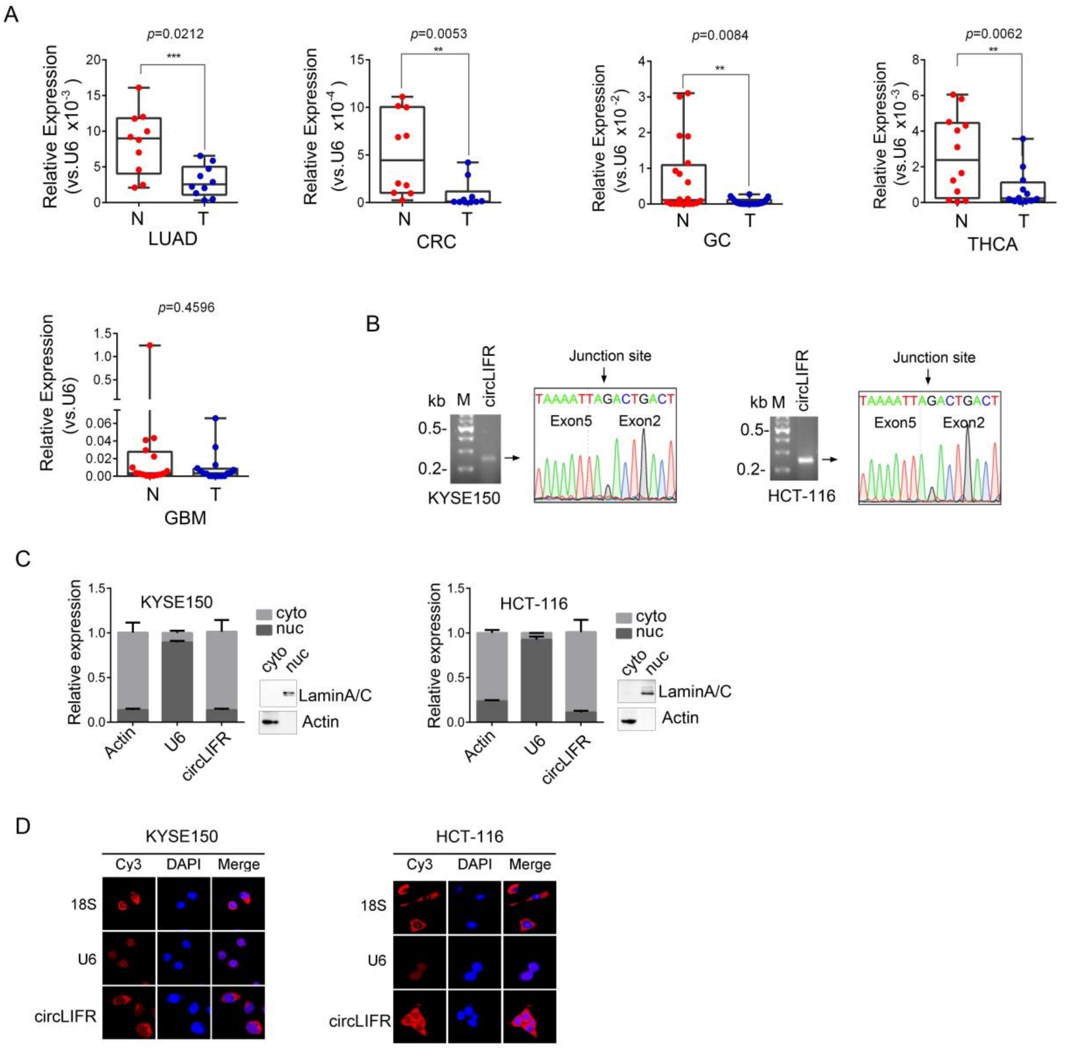

Fig S11. circLIFR expression in solid tumors and cells. **A** Quantification of circLIFR by RT-qPCR in solid tumors (LUAD, CRC, GC, THCA, GBM) and their matched adjacent normal tissues, and circLIFR expression was normalized to U6 mRNA levels. N, normal tissues; T, tumor tissues. **B I**dentification of the circularization site of circLIFR in KYSE150 and HCT-116 cells by RT-PCR and Sanger sequencing. **C**,**D** Cellular distribution of circLIFR in KYSE150 and HCT-116 cells, determined by cell nucleus/cytoplasm fractionation and RT-qPCR analyses (**C**) and fluorescence *in situ* hybridization (**D**). β-actin mRNA and U6 RNA represent cytoplasmic and nuclear RNAs, respectively. Western blotting confirmed the efficiency of nuclear/cytoplasmic isolation. Data are shown as the

mean ± SD.

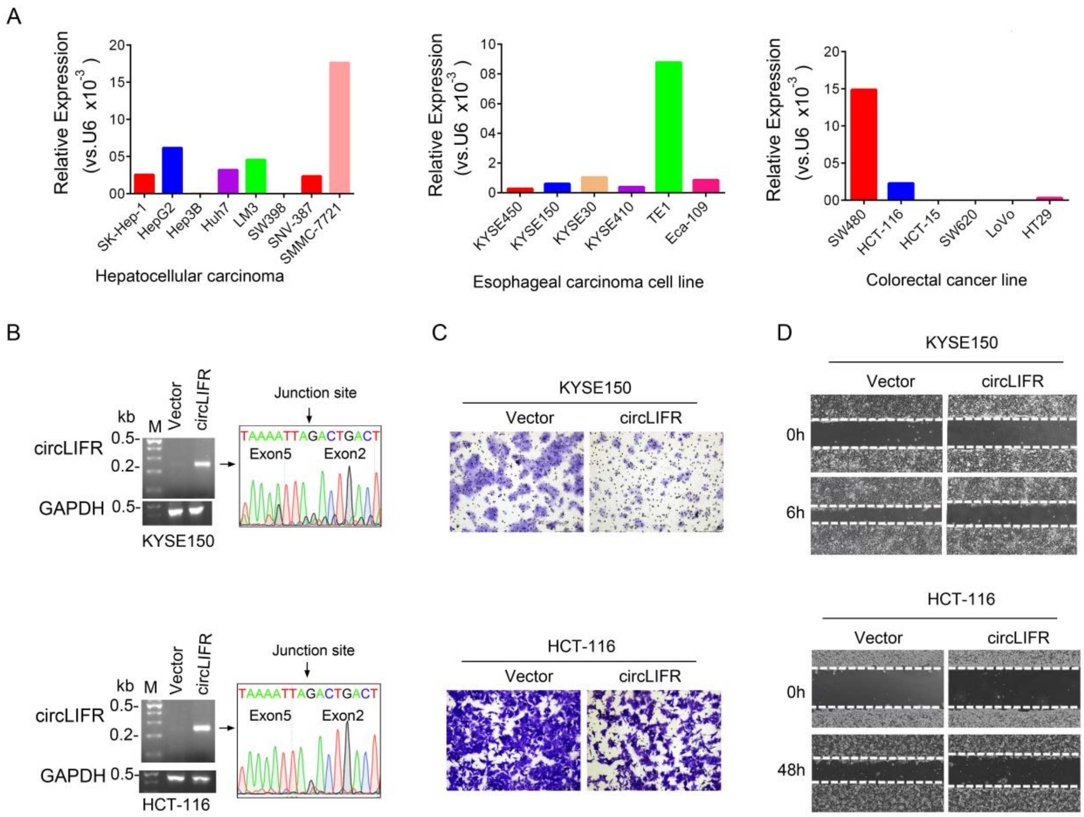

Fig S12. Effects of circLIFR on cell migration and motility in KYSE150 and HCT-116 cells. **A** Measurement of circLIFR expression in different cancer cells. U6 RNAs were used for normalization. **B** RT-PCR and Sanger sequencing showed that circLIFR was correctly overexpressed in different stable cancer cells. **C**,**D** Overexpression of circLIFR inhibited the migratory ability (**C**) and cell motility (**D**) of KYSE150 and HCT-116 cells.
